# Phylogeny Trumps Chemotaxonomy: A Case Study Involving *Turicella otitidis*

**DOI:** 10.3389/fmicb.2018.00834

**Published:** 2018-04-30

**Authors:** Inwoo Baek, Mincheol Kim, Imchang Lee, Seong-In Na, Michael Goodfellow, Jongsik Chun

**Affiliations:** ^1^School of Biological Sciences, Seoul National University, Seoul, South Korea; ^2^Institute of Molecular Biology and Genetics, Seoul National University, Seoul, South Korea; ^3^Division of Polar Life Sciences, Korea Polar Research Institute, Incheon, South Korea; ^4^Interdisciplinary Program in Bioinformatics, Seoul National University, Seoul, South Korea; ^5^School of Natural and Environmental Sciences, Newcastle University, Newcastle upon Tyne, United Kingdom

**Keywords:** *Turicella otitidis*, *Corynebacterium*, phylogenomic analysis, chemotaxonomy, mycolic acid, menaquinone

## Abstract

The genus *Turicella* was proposed to harbor clinical strains isolated from middle-ear fluids of patients with otitis media. 16S rRNA phylogeny showed that it belonged to the mycolic acid-containing actinobacteria, currently classified in the order *Corynebacteriales*, and was closely related to the genus *Corynebacterium*. A new genus was proposed for the organisms as unlike corynebacteria they lacked mycolic acids and had different menaquinones. Here, we carried out large-scale comparative genomics on representative strains of the genera *Corynebacterium* and *Turicella* to check if this chemotaxonomic classification is justified. Three genes that are known to play an essential role in mycolic acid biosynthesis were absent in *Turicella* and two other mycolate-less *Corynebacterium* spp., explaining the lack of mycolic acids resulted from the deletion of genes and does not confer any phylogenetic context. Polyphasic phylogenetic analyses using 16S rRNA, bacterial core genes and genes responsible for synthesizing menaquinones unequivocally indicate that *Turicella* is a true member of the genus *Corynebacterium*. Here, we demonstrate that menaquinone and mycolic acid that have been used as critical taxonomic markers should be interpreted carefully, particularly when genome-based taxonomy is readily available. Based on the phylogenetic analysis, we propose to reclassify *Turicella otitidis* as *Corynebacterium otitidis* comb. nov.

## Introduction

The order *Corynebacteriales* encompasses actinobacterial strains that are important in clinical, environmental and industrial microbiology (Lehmann and Neumann, 1896; Goodfellow and Jones, 2015). The taxon is well defined by the presence of mycolic acids, a special type of long chain fatty acid only found in members of this order. Mycolic acids are known to act as a cell wall permeability barrier that confers resistance to antibiotics and phagocytosis (Gebhardt et al., 2007). The length and number of double bonds in mycolic acids are used as important chemotaxonomic markers for distinguishing between genera classified in the *Corynebacteriales* (Bernard et al., 2010; Marrakchi et al., 2014) though some species in the order lack mycolic acids (Funke et al., 1994; Collins et al., 1998; Wiertz et al., 2013). Historically, the classification of mycolic acid-containing taxa was guided by a combination of chemotaxonomic and 16S rRNA gene sequence data; several new genera, including *Hoyosella* (Jurado et al., 2009) and *Lawsonella* (Bell et al., 2016) were recently described accordingly. However, in some cases, a lack of congruence between these sets of data required the use of addition taxonomic evidence to clarify the situation.

The classification of the genus *Turicella* is a case in point. The type and only species of this genus, *Turicella otitidis*, was initially proposed to harbor bacterial strains isolated from the ear of a patient with otitis media (Funke et al., 1994). In this study, the type strain of *T. otitidis* was recovered as a sister taxon to members of the genus *Corynebacterium* in the 16S rRNA gene tree while showing chemotaxonomic properties that differed from those of most *Corynebacterium* strains, notably the absence of mycolic acids and the presence of fully unsaturated menaquinones (MK-10 and MK-11), as opposed to dihydrogenated menaquinones [MK-8(H_2_) and MK-9(H_2_)]. However, later studies using more 16S rRNA gene sequences showed that *Turicella* formed a phyletic lineage within the *Corynebacterium* clade (Goyache et al., 2003; Hall et al., 2003).

Major fatty acid biosynthetic pathways, namely the FAS-I (Fatty acid synthesis-I) and FAS-II (Fatty acid synthesis-II) cycles, have been well documented for actinobacteria (Marrakchi et al., 2014). One multifunctional gene, 3-oxoacyl-ACP synthase (*fas*), participates in all reactions in the FAS-I cycle (Bloch and Vance, 1977). In contrast, four essential genes are involved in the FAS-II pathway, namely beta-ketoacyl-ACP synthase (*kasA*) (Bhatt et al., 2005), beta-ketoacyl-ACP reductase (*mabA*) (Parish et al., 2007), (3R)-hydroxy acyl-ACP dehydratase subunit B (*hadB*) (Brown et al., 2007; Sacco et al., 2007) and (NADH)-dependent trans-2-enoyl-ACP reductase (*inhA*) (Vilcheze et al., 2000). *Mycobacterium* spp. are known to have both the FAS-I and FAS-II pathways whereas most *Corynebacterium* spp. contain only the FAS-I pathway. It is reported that *Corynebacterium jeikeium* and *Corynebacterium urealyticum* have neither the FAS-I nor FAS-II cycle and obtain fatty acids from the exogenous environment (Tauch et al., 2005, 2008).

Mycolic acids are synthesized by a coupling of carboxylated fatty acids (α - branch) and meromycolic acids. Two kinds of carboxylases are involved; acyl-CoA carboxylase and acetyl-CoA carboxylase (Gande et al., 2007). The meromycolic acid is formed by the modification and activation of long-chain fatty acids. In *Mycobacterium tuberculosis*, several genes are known to participate in the modification of fatty acids, including desaturation (NADPH-dependent stearoyl-CoA 9-desaturase; *desA3*) (Cole et al., 1998), then, long-chain fatty acid AMP ligase (*fadD32*) leads to the synthesis of the meromycolic acid (Portevin et al., 2005). Finally, mycolic acids are formed by condensing two fatty acids by polyketide synthase 13 (*pks13*) (Portevin et al., 2004). It is known that three of these genes (*fadD32-pks13-accD4*) are located in a single operon which is essential for mycolic acid synthesis (Portevin et al., 2005); two beta common subunits of carboxylases (*accD4* and *accD5*) are also essential for mycolic acid synthesis (Gande et al., 2004).

Menaquinones are one of the major isoprenoid quinones required in bacterial electron transport systems (Nowicka and Kruk, 2010). The length and degree of saturation in isoprenoid chains of menaquinones are considered to be key taxonomic markers in the classification of *Actinobacteria* (Collins and Jones, 1981). Two major types of menaquinone biosynthetic pathways have been reported: the isochorismate and futalosine pathways (Seto et al., 2008). *Mycobacterium tuberculosis*, the most studied organism in the order *Corynebacteriales*, contains the former pathway; the component genes of this pathway were found in the genome of this organism (Dhiman et al., 2009). Upadhyay et al. (2015) found an enzyme, encoded by the menaquinone reductase (*menJ*) gene, confers the saturation of menaquinones of *M. tuberculosis*; the deletion of the *menJ* gene leads to the production of MK-9 instead of MK-9(H_2_).

In this study, we re-examine the confused taxonomic status of the genus *Turicella* using whole genome-based phylogeny and comparative genomics of genes responsible for synthesizing chemotaxonomic markers, namely fatty acids, mycolic acids, and menaquinones. On the basis of the evidence derived from the phylogenomic and comparative genomic analyses, it is proposed that *Turicella otitidis* be classified in the genus *Corynebacterium* as *Corynebacterium otitidis* comb. nov.

## Materials and Methods

### Genome Sequences and Identification of Genes

A set of 93 genome sequences includes type strains of 77 *Corynebacterium* spp., one *T. otitidis* (Brinkrolf et al., 2012), and 14 representative genera classified in the order *Corynebacteriales* (*Dietzia alimentaria*, *Gordonia bronchialis*, *Hoyosella altamirensis*, *Lawsonella clevelandensis*, *Millisia brevis*, *Mycobacterium tuberculosis*, *Nocardia asteroides*, *Rhodococcus rhodochrous*, *Segniliparus rotundus*, *Skermania piniformis*, *Smaragdicoccus niigatensis*, *Tomitella biformata*, *Tsukamurella paurometabola*, and *Williamsia muralis*). The type strain of *Pseudonocardia thermophila* was included as an outgroup. The whole-genome assemblies and their predicted CDS sequences were downloaded from the EzBioCloud database^1^ (Yoon et al., 2017). Genes related to the chemotaxonomic markers of the species were identified by a BLASTX search using BLAST+ v. 2.2.29 program (*E*-value cutoff = 1e-5, bit score cutoff = 100, identity cutoff = 30%; Rost, 1999) when compared against the reference gene sequences in the UniProt database^2^ (Apweiler et al., 2004). Genes of *M. tuberculosis* and *Corynebacterium glutamicum* were used as references for detecting mycolic acid biosynthetic genes (Ikeda and Nakagawa, 2003; Gande et al., 2007; Takeno et al., 2013; Marrakchi et al., 2014). Menaquinone biosynthesis genes in SwissProt, mainly *M. tuberculosis* and *Streptomyces coelicolor* (Seto et al., 2008), were used as references for the menaquinone biosynthesis pathway (Boutet et al., 2016). Description, abbreviation, and UniProt ID of all reference proteins are listed in Supplementary Table 1.

### Phylogenetic Analysis

The bacterial core gene-based phylogenetic analysis was carried out using the UBCG pipeline^3^ (Na et al., 2018). From the concatenated gene sequences extracted by UBCG, a maximum-likelihood phylogenetic tree was inferred using RAxML version 8.2.8 (Stamatakis, 2014) with the GTRGAMMA model and 100 bootstrap replications (Felsenstein, 1985). Another core-gene based genome-wide phylogenetic analysis was performed using PhyloPhlAn (Segata et al., 2013) based on CDS sequences of each genome downloaded in the previous step. The FastTree2 software implemented in this software was used to infer an approximated maximum-likelihood tree (Price et al., 2010; Liu et al., 2011). The 16S rRNA gene sequences of the same species were downloaded from the EzBioCloud database and aligned manually using secondary structure information by the EzEditor2 program (Jeon et al., 2014) and a maximum-likelihood phylogenetic tree reconstructed using RAxML with the GTRGAMMA model and 1,000 bootstrap replications (Felsenstein, 1985).

To infer the evolutionary history of genes involved in the biosynthesis of chemotaxonomic markers, a concatenated sequence alignment was generated from seven genes responsible for menaquinone biosynthesis (*menA* ∼*menG*), then used to elucidate the phylogenetic relationship using RAxML. Prior to concatenation, each gene was aligned separately using MAFFT software (v. 7.310) (Katoh and Standley, 2014). The Evolview web server^4^ was used to visualize the phylogenomic trees with the information on the presence of genes (He et al., 2016).

## Results

### Phylogeny of the Genus *Corynebacterium* and Related Taxa

**FIGURE 1:**
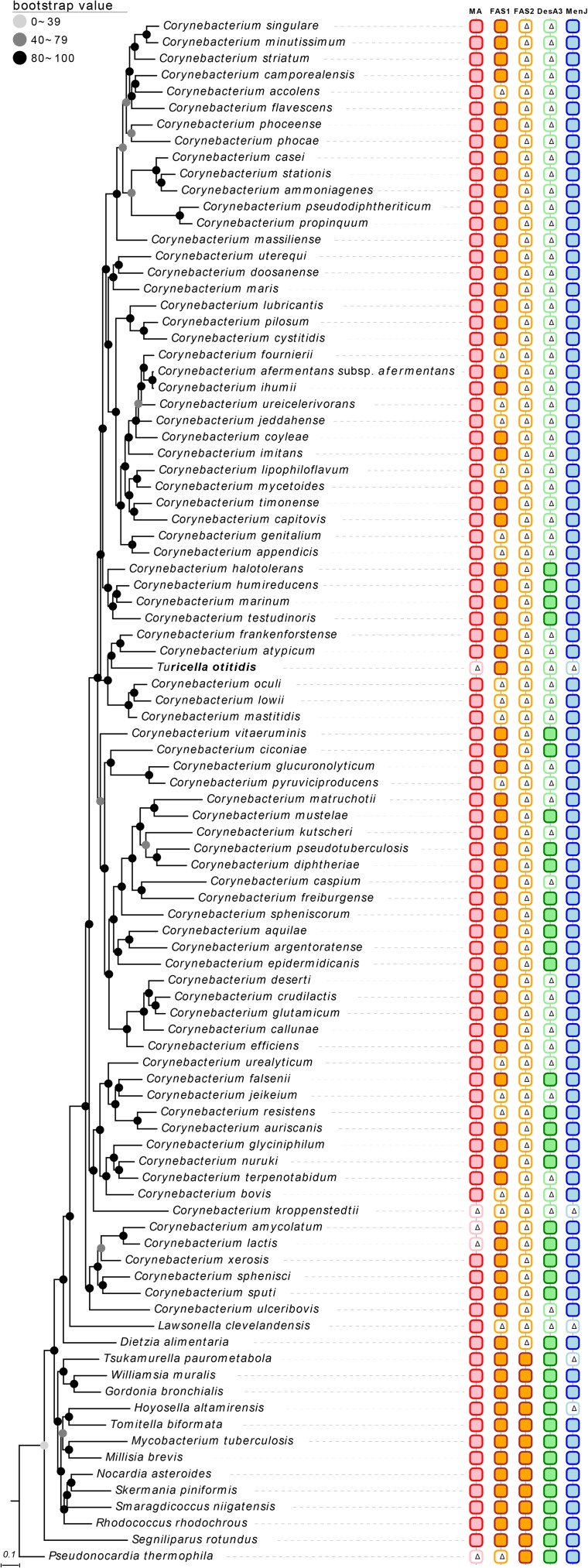
Genome-based phylogenetic tree reconstructed by the UBCG phylogenomics pipeline (Na et al., 2018) for *Turicella otitidis* and neighboring species. Bar in the below signifies substitution rate per site. Blocks in the column plots on the right side indicate the presence of each gene (or gene sets). Colored blocks indicate that the gene is present in the genome, and white blocks (with Δ sign) signify its absence. Numbers at the nodes indicate the bootstrap value of RAxML. Features and their corresponding genes: MA, mycolic acid biosynthetic genes; FAS1, fatty acid synthesis cycle 1 (*fas*); FAS2, fatty acid synthesis cycle 2 (*mabA*, *inhA*, *kasA*, and *hadB*); DesA3, NADPH-dependent stearoyl-CoA 9-desaturase; MenJ, menaquinone reductase.

**FIGURE 2:**
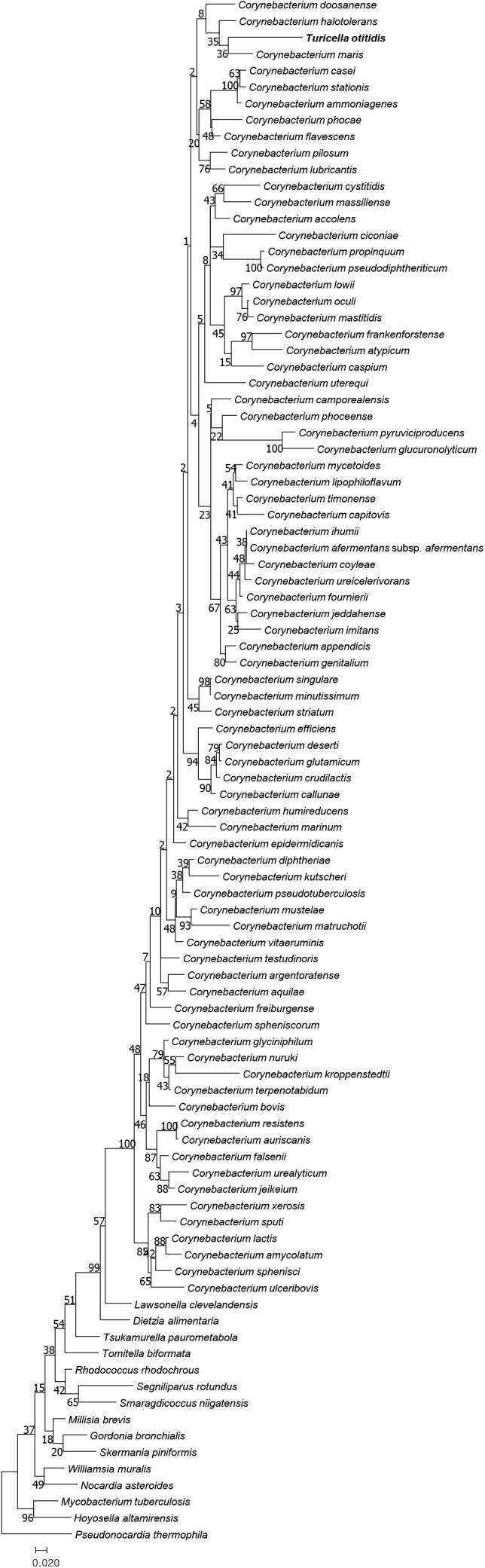
Phylogenetic tree reconstructed with 16S rRNA genes for *T. otitidis* and neighboring species. Bar indicates substitution rate per site. Numbers at the nodes are bootstrap values for 1,000 replicated subsamples expressed as percentage values.

The type strain of *T. otitidis* and those representing the *Corynebacterium* spp. formed a monophyletic clade in the phylogenomic trees inferred using both the UBCG (**Figure 1**) and PhyloPhlAn (Supplementary Figure 1) phylogenomic pipelines. The topology and phylogenetic relationships shown in the trees are in line with those from previous studies (Wu and Eisen, 2008; Wu et al., 2013), as well as from our analysis based on 16S rRNA phylogeny (**Figure 2**). It is clear that, unlike in the original study (Funke et al., 1994), *T. otitidis* does not form a sister taxon to the *Corynebacterium* clade but a phyletic lineage within the clade, indicating that it is a *bona fide* member of this genus.

### Genes Responsible for Fatty and Mycolic Acid Biosynthesis

**FIGURE 3:**
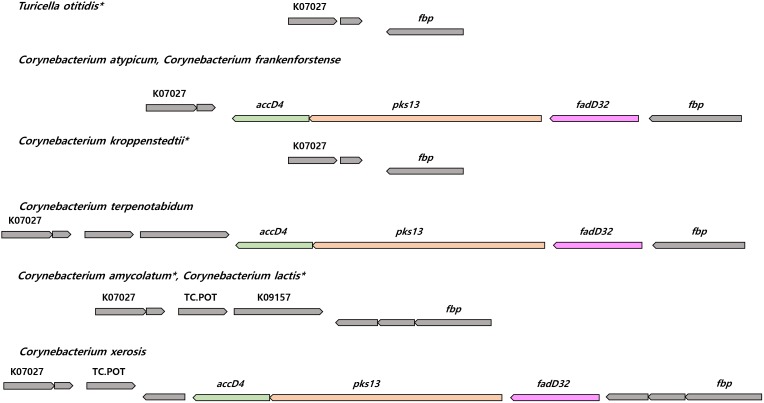
The synteny plot of gene families near *fadD32-pks13-accD4* operon site of species whose mycolic acid is absent and their phylogenetically neighboring species. Mycolic acid lacked species are marked with an asterisk. *accD4*, acyl-CoA carboxylase subunit beta 1; *pks13*, polyketide synthase; *fadD32*, long-chain-fatty-acid-AMP ligase; K07027, UPF0104 membrane protein; *fbp*, protein PS1; TC.POT, di/tripeptide transporter; K09157, UPF0210 protein.

The genomes of the type strain of *T. otitidis* and those of 61 out of the 77 *Corynebacterium* spp. contain the *fas* gene which expresses for the FAS-I pathway. Essential genes in the FAS-II pathway (*mabA*, *inhA*, *kasA*, and *hadB*) are absent from the genomes of *T. otitidis* and the *Corynebacterium* strains. The genomes of most *Corynebacteriales* species contain the four essential genes for the FAS-II cycle, the exceptions are *Dietzia alimentaria* and *Lawsonella clevelandensis* (Supplementary Tables 2, 3). *Dietzia alimentaria* lacks the *kasA* and *hadB* genes, and *L. clevelandensis* all of the FAS-I and FAS-II related genes. Comparative genomics and analysis of the synteny of genes responsible for mycolic acid biosynthesis suggest that *T. otitidis* and three mycolate-less *Corynebacterium* species (*Corynebacterium amycolatum*, *Corynebacterium kroppenstedtii*, and *Corynebacterium lactis*) lost the *fadD32-pks13-accD4* operon, located between the genes coding for protein PS1 and UPF0104 membrane protein in other species (**Figure 3**). They also lack *cmrA*, whereas all other *Corynebacterium* species have the necessary genes. In contrast, other carboxylase subunits that are known to be essential for mycolic acid biosynthesis (*accD2* and *accD3*) were found in the genomes of species that lack mycolic acids. Among the analyzed genomes, genes that are similar to those associated with mycolic acid modification (desaturation, cyclization, and methyl transfer) in *M. tuberculosis* were detected, though the sequence identity and the number of genes were not sufficient for further analysis. Exceptionally, genes homologous to *desA3*, a gene associated with the desaturation mycolic acids, were found in 23 of the *Corynebacterium* species; their sequence identity was around 50% compared to the reference (Supplementary Table 2). In contrast, two other kinds of putative desaturases (*desA1* and *desA2*) involved in the desaturation of mycolic acids were not detected in the *Corynebacterium* genomes.

### Genes Responsible for Menaquinone Biosynthesis

**FIGURE 4:**
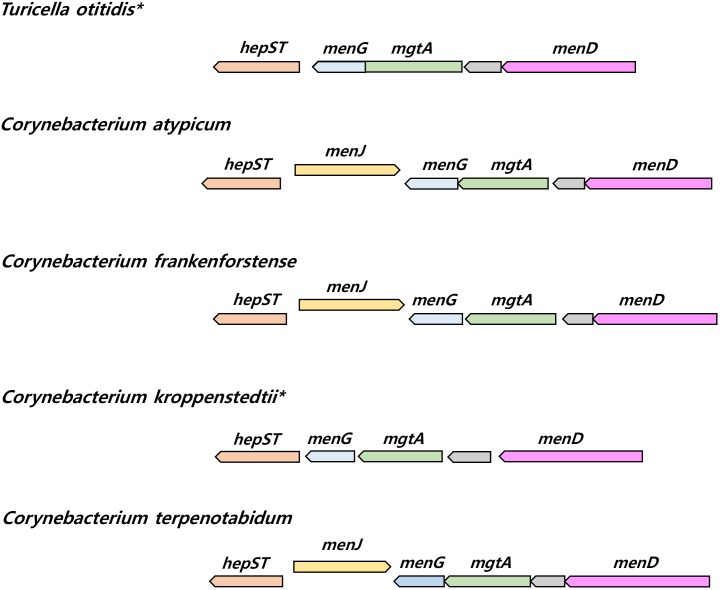
The synteny plot of gene families near *menJ* gene site of *T. otitidis* and related species. Species who lacked *menJ* gene are marked with an asterisk. *hepST*, geranyl pyrophosphate synthase; *menG*, demethylmenaquinone methyltransferase; *mgtA*, GDP-mannose-dependent alpha-mannosyltransferase; *menD*, 2-succinyl-5-enolpyruvyl-6-hydroxy-3-cyclohexene-1-carboxylate synthase.

The presence of genes responsible for menaquinone biosynthesis, including those participating in the elongation of isoprenoid side chains, was checked. Seven menaquinone biosynthetic genes (*menA* ∼*menG*), and a menaquinone side chain elongation gene (*uppS* and *hepST*) were common amongst the genomes of *Corynebacteriales* species including *T. otitidis*. On the other hand, a *menJ* gene implicated in menaquinone side chain saturation was not found in either *T. otitidis* or *C. kroppenstedtii*, though it was present in all of the other *Corynebacterium* genomes. This gene was also detected in the other *Corynebacteriales* species analyzed in this study, apart from those of *Hoyosella altamirensis*, *Lawsonella clevelandensis*, and *Tsukamurella paurometabola* which are reported to have fully unsaturated menaquinones (**Table 1**). The menaquinone composition of *C. kroppenstedtii* was not reported in the initial description (Collins et al., 1998). Comparative analysis of the gene synteny showed that the lack of *menJ* gene located between *hepST* and *menG* genes is apparent in both *T. otitidis* and *C. kroppenstedtii*, suggesting *C. kroppenstedtii* likely has fully unsaturated menaquinones (**Figure 4**).

**Table 1:** Differential chemotaxonomic characteristics of genera in the order *Corynebacteriales*.

Genus	TA	MA	DPG	PE	PI	PIM	MU	MK	G+C (%)
*Turicella*	+	-	ND	ND	ND	ND	ND	MK-10, MK-11	65–72
*Corynebacterium*	-^∗^	+^†^	+	-^§^	+	+	A	MK-8(H_2_), MK-9(H_2_)	46–74
*Dietzia*	+	+	+	+	+	+	A	MK-8(H_2_)	65.5–73
*Gordonia*	+	+	+	+	+	+	G	MK-9(H_2_)	63–69
*Hoyosella*	+	+	+	+	+	-	A	MK-8	49.3
*Lawsonella*	+	+	-	-	+	_	G	MK-9	58.6
*Millisia*	+	+	+	+	+	+	G	MK-8(H_2_)	64.7
*Mycobacterium*	+	+	+	+	+	+	G	MK-9(H_2_)	57–73
*Nocardia*	+	+	+	+	+	+	G	MK-8(H_4_, ω-cycl)	63–72
*Rhodococcus*	+	+	+	+	+	+	G	MK-8(H_2_)	63–73
*Segniliparus*	+	+	ND	ND	ND	ND	ND	ND	68–72
*Skermania*	+	+	+	+	+	+	G	MK-8(H_4_, ω-cycl)	67.5
*Smaragdicoccus*	-	+	-	+	+	+	G	SQA-8(H_4_, ω-cycl), SQB-8(H_4_, dicycl)	63.7
*Tomitella*	-	+	+	+	+	+	G	MK-9(H_2_)	59.3–71.6
*Tsukamurella*	+	+	+	+	+	+	G	MK-9	68–78
*Williamsia*	+	+	+	+	+	-	G	MK-9(H_2_)	64–65


*Data for Turicella and Tomitella were adapted from Funke et al. (1994) and Katayama et al. (2010), respectively. Data for other genera adapted from Bell et al. (2016). +, present; -, absent; TA, tuberculostearic acid; MA, mycolic acid; DPG, diphosphatidylglycerol; PE, phosphatidylethanolamine; PI, phosphatidylinositol; PIM, phosphatidylinositolmannosides; MU, muramic acid type; MK, menaquinone(s); A, acetylated; G, glycolated; ND, not determined. ^∗^Tuberculostearic acids were detected in some species (Collins et al., 1998; Kämpfer et al., 1999). ^†^C. amycolatum, C. caspium, C. ciconiae, C. kroppenstedtii, and C. lactis lack mycolic acids. ^§^ The presence of PE was reported at least for Corynebacterium bovis and C. urealyticum (Kämpfer et al., 1999).*

### Phylogenetic Analysis Using Concatenated Menaquinone Gene Sequences

**FIGURE 5:**
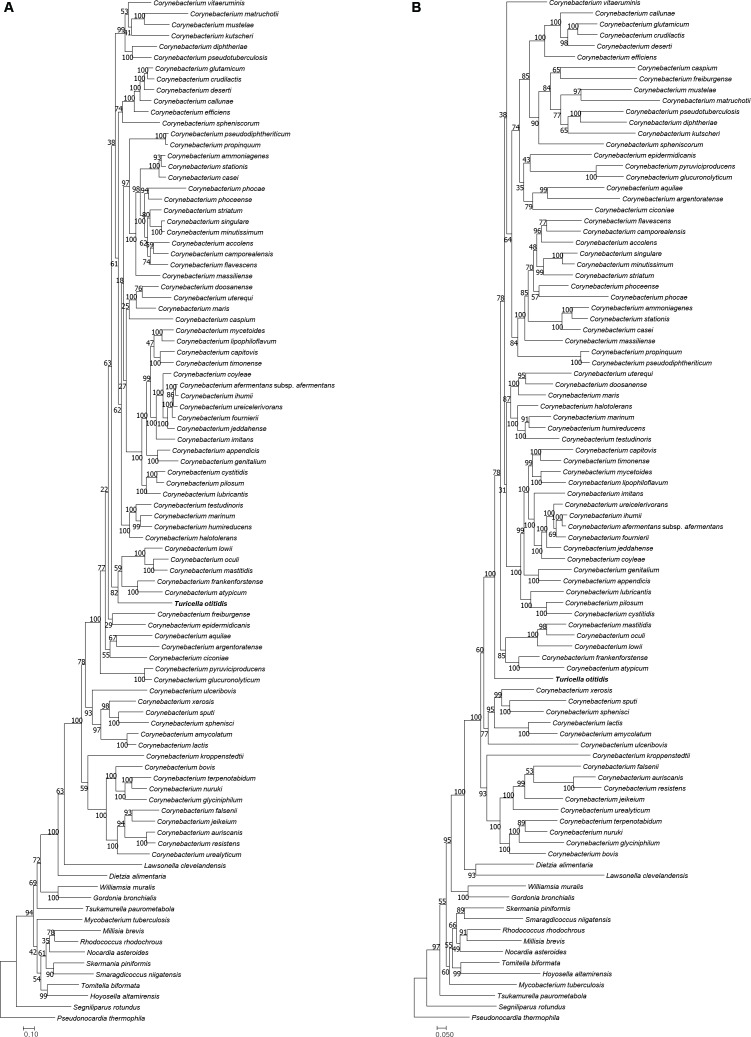
**(A)** Phylogenetic tree reconstructed with amino acid sequences of seven menaquinone biosynthetic genes for *T. otitidis* and its close species. **(B)** Phylogenetic tree reconstructed with nucleotide sequences of seven menaquinone biosynthetic gene families for *T. otitidis* and neighboring species. Bar indicates substitution rate per site. Numbers at the nodes are bootstrap values for 1,000 replicated subsamples expressed as a percentage value.

Gene-concatenated phylogenetic trees based on the seven genes (*menA* ∼*menG*) responsible for menaquinone biosynthesis were generated (**Figure 5**). As with the phylogenetic trees based on bacterial core and 16S rRNA genes, both nucleotide and amino acid gene concatenated trees support the inclusion of *T. otitidis* with the *Corynebacterium* species, even though the detail topologies within the evolutionary radiation occupied by the *Corynebacterium-Turicella* clade differ between trees.

## Discussion

The application of chemotaxonomic procedures led to marked improvements in prokaryotic systematics (Goodfellow et al., 2012) though little attention was paid to the confidence that could be placed on chemical characters. Experimental protocols used to acquire chemical data can be influenced by cultivation conditions, fatty acid profiles, for instance, are known to be sensitive to changes in temperature (Russell, 1984). Again, chemical markers generally are seen to be reliable, such as the presence or absence of mycolic acids in members of the order *Corynebacteriales* (Goodfellow and Jones, 2015), have been shown to be prone to experimental error. Thus, early reports that *Corynebacterium atypicum*, *Hoyosella altarmirensis*, and *Hoyosella subflava* lacked mycolic acids (Hall et al., 2003; Jurado et al., 2009; Wang et al., 2010) were found to be misplaced in light of later studies (Laneelle et al., 2012; Tippelt et al., 2014; Hamada et al., 2016; Li et al., 2016). Similarly, the report that the type strain of *Corynebacterium amycolatum* contained major amounts of dihydrogenated menaquinones with eight and nine isoprenoid units (Collins et al., 1988) was overturned by the discovery that this organism was rich in fully unsaturated menaquinones with nine isoprene units (Kämpfer et al., 1999; Jurado et al., 2009). In a broader context, it has been pointed out that the interpretation of polar lipid patterns based on two-dimensional thin-layer-chromatography is inherently subjective (Sutcliffe et al., 2012; Sutcliffe et al., 2013) while evidence that menaquinone composition can be significantly influenced when biomass is harvested at different stages of the growth cycle has generally been overlooked (Saddler et al., 1986). Such problems are compounded by the loss of single or small numbers of genes coding for chemotaxonomic features, as like the case of fatty acid biosynthesis gene loss in some *Corynebacterium* species (**Figure 1**), and by the fact that differences in a set of chemotaxonomic traits may be affected by one or a few genes and hence do not reflect phylogeny. For example, only *menJ* gene present species have saturated site in isoprenyl chain in their menaquinone (**Figure 1** and **Table 1**). Furthermore, there are two kinds of menaquinone biosynthetic pathways whose component genes are completely different (Seto et al., 2008), and the evolutionary history of those pathways was revealed (Zhi et al., 2014). Experimental chemotaxonomy cannot detect phylogenetic variance like this case. It is noteworthy that at present, we were not able to predict the isoprenyl chain length of menaquinones.

It is apparent from the present study that phylogenetic approaches can provide reliable ways of establishing whether *Corynebacteriales* strains are able to synthesis mycolic acids. It is particularly interesting that the gene set indispensable for the biosynthesis of mycolic acids (*fadD32-pks13-accD4 operon and cmrA*) is present in the genome of the *C. atypicum* and *Hoyosella* strains but have been lost from the genome of the *T. otitidis* strain. The genomes of the type strains of *Corynebacterium caspium* and *Corynebacterium ciconiae* also contain this gene set suggesting that they may synthesize mycolic acids (**Figure 1** and Supplementary Tables 2, 3) even though these strains have been reported to lack these components (Collins et al., 2004; Fernandez-Garayzabal et al., 2004). It is also interesting that the genes essential for the FAS-II fatty acid pathway (*hadB, inhA*, *kasA*, and *mabA*) are absent from the genome of *T. otitidis and Corynebacterium* strains but present in the genome of *Mycobacterium tuberculosis, Nocardia asteroides*, and *Rhodococcus erythropolis*. These observations suggest that genes involved in the same pathway evolve relative to one another. Furthermore, as in these cases, consecutive gene transfer or loss in a pathway can be used for the evidence for the classification when considering the parsimony of such events.

At present, genome-based phylogenies tend to be based on single-copy ubiquitous orthologous genes (Wu and Eisen, 2008; Wu et al., 2013). This practice yields consistent results albeit ones that may be biased as many of the target genes encode ribosomal proteins. Nevertheless, genes associated with key metabolic pathways can be used to supplement genome-derived phylogenetic inference. In this study, a phylogenetic tree based on concatenated sequences of seven menaquinone biosynthetic genes was used as a basis for phylogenetic re-identification given the significance of menaquinone composition in the identification of bacteria. Again, the current genomic analyses provided information on microbial structural phenotypes (**Figure 1**), without recourse to wet laboratory experimental data (**Table 1** and Supplementary Tables 2, 3). For example, the menaquinone profile of the type strain of *C. kroppenstedtii* is reported as MK-8 (Jurado et al., 2009), and the genome data can be used to infer that the major menaquinone of this organism, that is, fully unsaturated type given the absence of the *menJ* gene. It is noteworthy that the presence of genes or metabolic pathways derived from genomic information does not necessarily mean that it is always expressed to confer the phenotypes. However, this information can provide crucial clues about the phenotypes, including the chemotaxonomic markers.

At the time of the proposal of the genus *Turicella* (Funke et al., 1994), only three 16S rRNA gene sequences of the genus *Corynebacterium* were included in the phylogenetic analysis, leading to the conclusion of creating a new genus. The presence of fully unsaturated menaquinones in *Turicella* also supported this proposal. In this study, we showed that all aspects of phylogenetic evidence, including that based on menaquinone biosynthetic genes, indicate that *Turicella* is a genuine member of the genus *Corynebacterium* thereby supporting the view that phylogenomics provides more reliable data than chemotaxonomy in describing their evolutionary history and natural relationships, or that phylogenomics trumps chemotaxonomy. It can be inferred from the phenotypic inference of the genomes of *T. otitidis* that this organism has chemotaxonomic properties consistent with its classification in the genus *Corynebacterium*, notably the presence of genes associated with the FAS-II pathway. Consequently, it is proposed that *T. otitidis* be reclassified as *Corynebacterium otitidis* comb. nov.

### Emended Description of the Genus *Corynebacterium* Lehmann and Neumann (1896)

The description is that given by Bernard et al. (2010) with the following changes. Most species produce menaquinones with partially saturated isoprenoid chains [mainly MK-8(H2) and/or MK-9(H2)], but for some species the fully unsaturated menaquinones MK-8, MK-9, MK-10, and MK-11 were found.

### Description of *Corynebacterium otitidis* (Funke et al., 1994) comb. nov.

*Corynebacterium otitidis* (o.ti’ti.dis. L. gen. n. of *otitis*, inflammation of the ear). Basonym: *Turicella otitidis* Funke et al. (1994). This description is the same as that given by Funke et al. (1994). The type strain is 234/92^T^ (=DSM 8821^T^ = ATCC 51513^T^).

## Author Contributions

The idea for this study was conceived by JC and IB, who was also charged with supervising the whole project. Data collecting and reference searching, analyzing genomic data, and writing a draft of the manuscript scheme were done by IB. Phylogenomic trees were reconstructed by IB, IL, and S-IN, with applying the mechanisms and proposed the idea. The manuscript was written, curated, and confirmed by IB, MK, MG, and JC.

## Conflict of Interest Statement

The authors declare that the research was conducted in the absence of any commercial or financial relationships that could be construed as a potential conflict of interest.

## Footnotes

1 https://www.ezbiocloud.net/

2 http://www.uniprot.org/

3 https://www.ezbiocloud.net/tools/ubcg

4 http://evolgenius.info/evolview/
