# Supplementary material for: Phylogeny Trumps Chemotaxonomy: A Case Study Involving Turicella otitidis
Source: Front Microbiol. 2018 Apr 30;9:834. doi: 10.3389/fmicb.2018.00834 (PMC5936774; doi:10.3389/fmicb.2018.00834)
Supplement: Supplementary file 1 [file Table_1.pdf]

Supplementary Table 1. UniProt Accession numbers for the reference sequences in the analysis

Pathway	Abbreviation	Description of Protein	UniProt-ID
Fatty Acid Synthesis-I	*fas*	3-oxoacyl-ACP synthase	P95029(MT), Q8NS46(CG)
Fatty Acid Synthesis-II	*fabD*	Malonyl CoA-acyl carrier protein transacylase	**P9WNG5(MT)**
	fabH	3-oxoacyl-[acyl-carrier-protein] synthase 3	**P9WNG3(MT)**
	kasA	3-oxoacyl-[acyl-carrier-protein] synthase 1	**P9WQD9(MT)**
	kasB	3-oxoacyl-[acyl-carrier-protein] synthase 2	**P9WQD7(MT)**
	mabA	3-oxoacyl-[acyl-carrier-protein] reductase	**P9WGT3(MT)**
	hadA	(3R)-hydroxyacyl-ACP dehydratase subunit HadA	**P9WFK1(MT)**
	hadB	(3R)-hydroxyacyl-ACP dehydratase subunit HadB	I6WYY7(MT)
	hadC	(3R)-hydroxyacyl-ACP dehydratase subunit HadC	**P9WFJ9(MT)**
	inhA	Enoyl-[acyl-carrier-protein] reductase	**P9WGR1(MT)**
Mycolic Acid Biosynthesis: Carboxylation	*accD6*	Acetyl-CoA carboxylase beta subunit	**P9WQH5(MT)**
	accD4	Acyl-CoA carboxylase subunit beta 1	O53578(MT)
	accD5	Acyl-CoA carboxylase subunit beta 2	**P9WQH7(MT)**
	accA3	Acyl/Acetyl-CoA carboxylase subunit alpha	P96890(MT)
	accE	Acyl/Acetyl-CoA carboxylase subunit epsilon	P96886(MT)
Mycolic Acid Biosynthesis: Condensation	*pks13*	Polyketide synthase	I6X8D2(MT), Q8NLR7(CG)
Mycolic Acid Biosynthesis: Activation	*fadD32*	Long-chain-fatty-acid--AMP ligase	**O53580(MT),** Q8NLR6(CG)
Mycolic Acid Biosynthesis: Reduction	*cmrA*	Probable short-chain type dehydrogenase/reductase	I6Y9I3(MT), Q8NMU2(CG)
Mycolic Acid Biosynthesis: Transport	*mmpL3*	Trehalose monomycolate exporter	**P9WJV5(MT)**, Q8NLS1(CG)
Mycolic Acid Cyclization, Methylation	*cmaA1*	Cyclopropane mycolic acid synthase 1	**P9WPB7(MT)**
	cmaA2	Cyclopropane mycolic acid synthase 2	**P9WPB5(MT)**
	umaA1	Possible mycolic acid synthase	**Q6MX39(MT)**
	pcaA	Cyclopropane mycolic acid synthase 3	**P9WPB3(MT)**
	mmaA1	Mycolic acid methyltransferase	**P9WPB1(MT)**
	mmaA2	Cyclopropane mycolic acid synthase	**Q79FX6(MT)**
	mmaA3	Methoxy mycolic acid synthase	**P0CH91(MT)**
	mmaA4	Hydroxymycolate synthase	**Q79FX8(MT)**
Mycolic Acid Desaturation	*desA1*	Putative acyl-desaturase	**P9WNZ7(MT)**
	desA2	Putative acyl-desaturase	**P9WNZ5(MT)**
	desA3	NADPH-dependent stearoyl-CoA 9-desaturase	**P9WNZ3(MT)**
Menaquinone Biosynthesis Pathway: Classical	*menA*	1,4-dihydroxy-2-naphthoate octaprenyltransferase	**P9WIP3(MT)**
	menB	1,4-dihydroxy-2-naphthoyl-CoA synthase	**P9WNP5(MT)**
	menC	o-succinylbenzoate synthase	**P9WJP3(MT)**
	menD	2-succinyl-5-enolpyruvyl-6-hydroxy-3-cyclohexene-1-carboxylate synthase	**P9WK11(MT)**
	menE	2-succinylbenzoate--CoA ligase	**P9WQ39(MT)**
	menF	Isochorismate synthase	**P9WFW9(MT)**
	menG	Demethylmenaquinone methyltransferase	**P9WFR3(MT)**
Menaquinone Biosynthesis Pathway: Futalosine	*mqnA*	Chorismate dehydratase	**Q9L0T8(SCO), A1W0R9(CJ), Q5SK49(TT)**
	mqnB	Futalosine hydrolase	**Q9KXN0(SCO), Q5SKT7(TT), A0LR22(ACE)**
	mqnC	Cyclic dehypoxanthine futalosine synthase	**Q9XAP2(SCO), Q9K864(BH)**
	mqnD	1,4-dihydroxy-6-naphtoate synthase	**Q9KXN1(SCO), Q5SI12(TT)**
	mqnE	Aminodeoxyfutalosine synthase	**Q8CJT5(SCO), Q5SK48(TT)**
	SCO4490	Putative decarboxylase	Q9KYP3(SCO)
	SCO4491	Putative octaprenyltransferase	Q9KYP2(SCO)
	ubiX	Flavin prenyltransferase	**Q9KYP1(SCO)**
Menaquinone Isoprenyl Chain Elongation	*uppS*	Decaprenyl diphosphate synthase	**P9WFF7(MT)**
	hepST	Geranylgeranyl pyrophosphate synthase	Q8NT37(CG)
Menaquinone Isoprenyl Chain Saturation	*menJ*	Menaquinone reductase	**P9WNY9(MT)**
