# Supplementary material for: Phylogeny Trumps Chemotaxonomy: A Case Study Involving Turicella otitidis
Source: Front Microbiol. 2018 Apr 30;9:834. doi: 10.3389/fmicb.2018.00834 (PMC5936774; doi:10.3389/fmicb.2018.00834)
Supplement: Supplementary file 2 [file Table_2.PDF]

Supplementary Table 2. Gene detected in Corynebacterium genomes associated with the biosynthesis of mycolic acids, and menaquinones.

Assembly Accession	Species name	Strain name	Mycolic acid Biosynthesis																														Menaquinone biosynthesis - classical pathway							Elongation		Saturation	Menaquinone biosynthesis - futalosine pathway							
			fas	*fabD*	*fabH*	*kasA*	*kasB*	*mabA*	*hadA*	*hadB*	*hadC*	*inhA*	*accD6*	*accA3*	*accE*	*accD4*	*accD5*	*pks13*	*fadD32*	*cmrA*	*mmpL3*	*cmaA1*	*cmaA2*	*umaA1*	*pcaA*	*mmaA1*	*mmaA2*	*mmaA3*	*mmaA4*	*desA1*	*desA2*	*desA3*	*menA*	*menB*	*menC*	*menD*	*menE*	*menF*	*menG*	*uppS*	*hepST*	*menJ*	*mqnA*	*mqnB*	*mqnC*	*mqnD*	*mqnE*	*SCO4490*	*SCO4491*	*ubiX*
GCA_000159115.1	*Corynebacterium accolens*	ATCC 49725	ND	ND	ND	31.82	ND	34.43	ND	ND	ND	ND	45.47	74.28	34.72	77.33	50.28	64.84	57.35	65.04	66.9	34.43	34.84	36.26	33.7	34.8	34.07	33.1	34.04	ND	ND	ND	56.88	70.21	50.74	40.11	44.38	44.26	69.2	60.57	64.53	49.21	ND	ND	ND	ND	ND	ND	ND	ND
GCA_900156035.1	*Corynebacterium afermentans* subsp. *afermentans*	DSM 44280	56.92	ND	ND	31.58	ND	ND	ND	ND	ND	ND	72.69	72.31	ND	75.83	61.77	63	53.91	64.66	63.75	33.69	33.45	35.14	31.99	31.88	31.14	30.88	ND	ND	ND	ND	56.25	78.36	57.59	42.15	46.24	43.35	65.38	58.75	61.78	49.74	ND	ND	ND	ND	ND	ND	ND	ND
GCA_001941425.1	*Corynebacterium ammoniagenes*	DSM 20306	60.55	ND	ND	ND	ND	34.4	ND	ND	ND	ND	82.32	77.16	41.38	77.04	67.49	65.26	64.81	66.17	62.55	33.57	32.52	36.63	33.94	33.7	32.62	33.22	33.21	ND	ND	ND	56.27	77.56	53.27	38.22	46.49	43.41	69.47	62.03	65.43	50.82	ND	ND	ND	ND	ND	ND	ND	ND
GCA_000755185.1	*Corynebacterium amycolatum* *	ATCC 6931†	48.46	ND	ND	ND	ND	ND	ND	ND	ND	ND	72.11	80.95	ND	ND	84.17	ND	30.61	ND	ND	31.87	33.92	37.55	31.09	32.73	31.02	32.87	32.62	ND	ND	50.81	55.75	74.84	57.4	43.18	40.86	44.66	65.2	63.29	59.35	50.76	ND	ND	ND	ND	ND	ND	ND	ND
GCA_900156665.1	*Corynebacterium appendicis*	DSM 44531	ND	ND	ND	30.1	30.1	ND	ND	ND	ND	ND	45.8	70.27	38.75	77.3	49.91	62.63	57.77	68.8	61.68	31.77	34.49	35.51	32.72	31.52	ND	30.96	30.64	ND	ND	ND	55.48	78.78	53.85	42.28	43.63	45.14	66.08	58.65	59.82	50.13	ND	ND	ND	ND	ND	ND	ND	ND
GCA_001941445.1	*Corynebacterium aquilae*	S-613	51.07	ND	ND	ND	30.93	33.91	ND	ND	ND	ND	83.18	83.64	46.91	80.51	88.75	66.2	65.15	65.41	70.24	ND	ND	ND	ND	ND	ND	ND	ND	ND	ND	52.91	54.84	76.28	51.29	40.7	46.72	42.19	69.3	58.51	67.69	46.3	ND	ND	ND	ND	ND	ND	ND	ND
GCA_000590555.1	*Corynebacterium argentoratense*	DSM 44202	50.66	ND	ND	ND	ND	ND	ND	ND	ND	ND	82.82	84.82	45.78	81.52	88.93	67.59	65.14	65.04	66.62	ND	ND	ND	ND	ND	ND	ND	ND	ND	ND	51.32	54.75	79.54	49.57	41.71	46.69	41.58	66.67	56.09	69.81	52.69	ND	ND	ND	ND	ND	ND	ND	ND
GCA_000732945.1	*Corynebacterium atypicum*	R2070	48.79	ND	ND	ND	ND	ND	ND	ND	ND	ND	46.95	67.58	ND	76.18	55.45	64.02	60.13	71.8	73.06	34.19	33.57	37.36	33.82	31.87	31.41	31.75	32.49	ND	ND	ND	58.93	80.13	55.18	44.21	44.84	45.95	71.62	59.07	63.58	46.78	ND	ND	ND	ND	ND	ND	ND	ND
GCA_000767255.1	*Corynebacterium auriscanis*	CIP 106629	49.43	ND	ND	ND	ND	33.16	ND	ND	ND	ND	46.7	70.82	ND	70.51	49.07	61.21	60.07	56.44	63.94	ND	ND	ND	ND	ND	ND	ND	ND	ND	ND	45.73	59.79	78.14	45.95	40.66	38.53	41.96	68.75	61.54	60.49	48.33	ND	ND	ND	ND	ND	ND	ND	ND
GCA_000183325.2	*Corynebacterium bovis*	DSM 20582	ND	ND	ND	ND	ND	ND	ND	ND	ND	ND	46.74	73.85	32.81	69.34	48.83	64.26	58.93	55.51	64.61	ND	ND	31.51	ND	ND	ND	ND	ND	ND	ND	ND	57.4	79.53	55.83	61.94	45.85	40.37	70.48	61.14	62.65	52.74	ND	ND	ND	ND	ND	ND	ND	ND
GCA_000344785.1	*Corynebacterium callunae*	DSM 20147	90.77	ND	ND	ND	ND	ND	ND	ND	ND	ND	94.66	92.06	57.32	91.09	79.54	85.06	85.23	86.09	85.71	34.06	34.27	35.52	33.98	34.07	32.43	32.13	34.46	ND	ND	ND	57.44	74.21	51.63	40.53	42.94	43.56	70.18	65.02	89.43	50.4	ND	ND	ND	ND	ND	ND	ND	ND
GCA_000980815.1	*Corynebacterium camporealensis*	DSM 44610	47.84	ND	ND	30.42	ND	35.18	ND	ND	ND	ND	79.56	73.77	47.89	77.06	68.05	65.05	63.68	66.92	68.35	ND	ND	ND	ND	ND	ND	ND	ND	ND	ND	ND	54.36	72.76	49.71	39.89	43.78	45.75	67.11	60.41	63.64	49.74	ND	ND	ND	ND	ND	ND	ND	ND
GCA_000372085.1	*Corynebacterium capitovis*	DSM 44611	61.71	ND	ND	30.89	ND	32.24	ND	ND	ND	ND	74.36	73.46	43.28	76.8	66.35	63.13	54.81	67.29	62.18	ND	ND	ND	ND	ND	ND	ND	ND	ND	ND	ND	56.1	74.68	56.56	41.71	47.7	44.08	68.86	53.36	64.09	50.41	ND	ND	ND	ND	ND	ND	ND	ND
GCA_000550785.1	*Corynebacterium casei*	LMG S-19264	46.48	ND	ND	ND	ND	ND	ND	ND	ND	ND	81.95	77.66	40.23	75.88	67.49	64.16	65.71	66.92	66.67	ND	ND	ND	ND	ND	ND	ND	ND	ND	ND	ND	54.48	76.6	53.69	37.84	45.8	43.68	69.03	61.86	64.2	47.01	ND	ND	ND	ND	ND	ND	ND	ND
GCA_000379705.1	*Corynebacterium caspium**	DSM 44850	61.83	ND	ND	ND	30.53	31.69	ND	ND	ND	ND	75.85	73.23	ND	78.52	64.3	66.63	58.92	71.7	69.42	ND	ND	ND	ND	ND	ND	ND	ND	ND	ND	ND	58.19	79.02	52.98	38.12	42.2	44.72	68.42	60.76	64.92	50.13	ND	ND	ND	ND	ND	ND	ND	ND
GCA_000372385.1	*Corynebacterium ciconiae**	DSM 44920	45.1	ND	ND	ND	ND	31.22	ND	ND	ND	ND	77.84	83.45	62.86	78.52	86.51	64.77	65.14	74.06	66.8	ND	ND	ND	ND	ND	ND	ND	ND	ND	ND	48.67	57.29	76.3	54.18	39.57	46.04	44.72	70.8	63.37	61.54	50.26	ND	ND	ND	ND	ND	ND	ND	ND
GCA_900105505.1	*Corynebacterium coyleae*	DSM 44184	57.11	ND	ND	ND	ND	33.88	ND	ND	ND	ND	73.33	73.59	ND	77	62.67	64.15	54.22	68.05	63.53	ND	ND	ND	ND	ND	ND	ND	ND	ND	ND	ND	51.92	78.36	55.96	42.34	44.24	42.78	66.67	58.75	60.58	48.82	ND	ND	ND	ND	ND	ND	ND	ND
GCA_001643015.1	*Corynebacterium crudilactis*	JZ16	94.98	ND	ND	ND	ND	35.83	ND	ND	ND	ND	97.97	95.6	82.93	96.9	95.03	91.44	93.45	92.86	87.67	ND	ND	ND	ND	ND	ND	ND	ND	ND	ND	ND	55.4	72.64	51.79	40.77	41.3	42.33	69.74	65.84	88	50.66	ND	ND	ND	ND	ND	ND	ND	ND
GCA_900111265.1	*Corynebacterium cystitidis*	DSM 20524	60.6	ND	ND	ND	31.48	33.61	ND	ND	ND	ND	85.27	75.68	36.76	77.58	69	62.22	63.68	65.91	59.57	39.16	ND	39.42	ND	ND	42.55	38.1	38.36	ND	ND	ND	55.52	75.48	55.7	43.75	40.91	44.89	67.98	59.83	66.87	52.56	ND	ND	ND	ND	ND	ND	ND	ND
GCA_001277995.1	*Corynebacterium deserti*	GIMN1.010	93.5	ND	ND	ND	ND	39.67	ND	ND	ND	ND	97.61	94.25	74.39	93.22	82.31	89.39	90.57	92.48	89.77	32.72	35.66	35.9	33.2	33.7	30.88	31.77	33.57	ND	ND	ND	55.75	73.9	53.57	40.96	42.19	43.72	70.18	64.61	90.29	50.53	ND	ND	ND	ND	ND	ND	ND	ND
GCA_001457455.1	*Corynebacterium diphtheriae*	NCTC 11397	50.55	ND	ND	ND	ND	36.51	ND	ND	ND	ND	82.14	85.14	48.61	81.36	88.58	68.34	68.7	74.06	70.33	ND	ND	ND	ND	ND	ND	ND	ND	ND	ND	53.68	56.63	76.95	50.91	40.29	44.35	44.17	62.5	60.84	70.25	53.3	ND	ND	ND	ND	ND	ND	ND	ND
GCA_000767055.1	*Corynebacterium doosanense*	CAU 212	62.65	ND	ND	ND	ND	ND	ND	ND	ND	ND	80.48	76.65	ND	77.73	65.97	65.61	67.11	70.3	66.93	ND	ND	ND	ND	ND	ND	ND	ND	ND	ND	ND	58.1	74.92	52.35	43.22	46.34	46.87	66.96	59.75	61.75	50.13	ND	ND	ND	ND	ND	ND	ND	ND
GCA_000011305.1	*Corynebacterium efficiens*	YS-314	85.21	ND	ND	ND	ND	33.88	ND	ND	ND	ND	97.24	90.54	62.2	89.36	91.59	84.45	86.85	84.53	82.97	ND	ND	ND	ND	ND	ND	ND	ND	ND	ND	ND	60.29	72.59	52.52	40.82	45.32	45.53	70.43	63.79	82.29	48.48	ND	ND	ND	ND	ND	ND	ND	ND
GCA_001021025.1	*Corynebacterium epidermidicanis*	DSM 45586	51.58	ND	ND	ND	ND	33.05	ND	ND	ND	ND	79.08	85.33	47.14	81.04	88.91	69.4	62.35	72.18	68.63	ND	ND	ND	ND	ND	ND	ND	ND	ND	ND	53.92	59.04	75.39	52.23	41.09	43.94	41.47	65.93	63.33	64.42	50.55	ND	ND	ND	ND	ND	ND	ND	ND
GCA_000525655.1	*Corynebacterium falsenii*	DSM 44353	47.23	ND	ND	ND	ND	ND	ND	ND	ND	ND	47.24	72.82	ND	71.01	50.1	61.06	58.05	58.94	63.99	ND	ND	ND	ND	ND	ND	ND	ND	ND	ND	49.38	58.06	75.86	40.74	41.59	38.31	42.08	67.54	59.6	60.8	48.84	ND	ND	ND	ND	ND	ND	ND	ND
GCA_001941465.1	*Corynebacterium flavescens*	OJ8	60.91	ND	ND	ND	30.05	34.44	ND	ND	ND	ND	81.95	77.16	49.3	76.22	67.12	65.44	64.53	65.02	65.58	ND	ND	ND	ND	ND	ND	ND	ND	ND	ND	ND	58.87	72.09	52.35	40.03	45.28	42.55	70.18	59.32	65.05	49.74	ND	ND	ND	ND	ND	ND	ND	ND
GCA_900176865.1	*Corynebacterium fournierii*	Marseille-P2948	ND	ND	ND	ND	ND	31.8	ND	ND	ND	ND	46.96	71.69	ND	75.24	49.05	66.67	54.32	64.66	62.73	33.69	33.1	35.14	31.62	31.88	31.14	30.88	ND	ND	ND	ND	55.68	78.36	54.74	41.87	43.48	41.78	66.67	57.5	61.49	49.47	ND	ND	ND	ND	ND	ND	ND	ND
GCA_001941485.1	*Corynebacterium frankenforstense*	ST18	61.49	ND	ND	ND	ND	34.57	ND	ND	ND	ND	75.68	75.63	ND	79.01	66.22	64.26	67.62	76.98	66.75	32.35	32.06	35.16	32.72	31.87	31.05	30.6	32.13	ND	ND	ND	61.31	74.52	50.62	42.78	44.05	45.3	71.18	59.83	63.05	47.38	ND	ND	ND	ND	ND	ND	ND	ND
GCA_000428805.1	*Corynebacterium freiburgense*	DSM 45254	50.13	ND	ND	ND	ND	32.49	ND	ND	ND	ND	82.32	84.12	50	79.84	89.44	68.19	67.34	71.32	64.64	ND	ND	ND	ND	ND	ND	ND	ND	ND	ND	50.92	55.17	76.87	53.61	41.06	40.38	41.89	67.4	65.53	67.95	50.67	ND	ND	ND	ND	ND	ND	ND	ND
GCA_000143825.1	*Corynebacterium genitalium*	ATCC 33030	ND	ND	ND	ND	30.5	31.17	ND	ND	ND	ND	46.27	68.58	42.19	77.67	51.13	62.19	56.12	66.92	62.76	31.41	32.75	34.06	31.39	30.43	ND	ND	31.05	ND	ND	ND	55.94	77.1	49.41	42.19	45.53	42.08	62.56	57.83	62.73	49.48	ND	ND	ND	ND	ND	ND	ND	ND
GCA_000159595.1	*Corynebacterium glucuronolyticum*	ATCC 51866	55.16	ND	ND	ND	ND	ND	ND	ND	ND	ND	69.49	78.89	41.98	78.14	84.4	61.62	56.97	76.14	69.05	ND	ND	ND	ND	ND	ND	ND	ND	ND	ND	ND	49.29	80.72	49.69	40.29	46.99	46.74	65.64	63.56	64.4	46.97	ND	ND	ND	ND	ND	ND	ND	ND
GCA_000011325.1	*Corynebacterium glutamicum*	ATCC 13032	99.97	ND	ND	ND	ND	33.08	ND	ND	ND	ND	100	99.83	100	99.81	100	100	99.83	100	99.87	32.61	33.22	35.07	32.43	33.7	ND	30.42	32.18	ND	ND	ND	55.94	73.58	53.73	41.95	42.45	43.46	68.86	64.61	100	50	ND	ND	ND	ND	ND	ND	ND	ND
GCA_000626675.1	*Corynebacterium glyciniphilum*	AJ 3170	48.06	ND	ND	ND	ND	39.13	ND	ND	ND	ND	51.41	68.87	ND	68.55	56.53	60.38	56.84	56.98	64.35	34.19	30.32	33.46	34.07	32.71	32.22	32.84	33.21	ND	ND	50.13	58.48	73.33	50.75	43.36	42.91	40.24	70.09	62.4	61.28	51.27	ND	ND	ND	ND	ND	ND	ND	ND
GCA_000341345.1	*Corynebacterium halotolerans*	YIM 70093	51.24	ND	ND	ND	ND	31.98	ND	ND	ND	ND	87.29	79.02	40.54	82.56	67.23	71.6	67.68	72.93	76.45	31.99	34.94	36.5	32.82	31.5	31.8	30.51	32.26	ND	ND	53.24	58.33	79.8	54.3	41.51	45.8	45.26	69.83	64.26	70.06	51.48	ND	ND	ND	ND	ND	ND	ND	ND
GCA_000819445.1	*Corynebacterium humireducens*	DSM 45392	50.65	ND	ND	ND	ND	38.46	ND	ND	ND	ND	86.74	81.05	ND	80.98	68.24	70.62	70	70.3	77.34	ND	ND	ND	ND	ND	ND	ND	ND	ND	ND	51.19	56.74	76.57	50.46	40.91	44.29	45.18	68.42	64.56	67.89	48.96	ND	ND	ND	ND	ND	ND	ND	ND
GCA_000403725.1	*Corynebacterium ihumii*	GD7	56.74	ND	ND	ND	ND	ND	ND	ND	ND	ND	72.88	72.53	ND	76.02	61.58	62.63	53.91	64.66	63.89	ND	ND	ND	ND	ND	ND	ND	ND	ND	ND	ND	55.17	78.36	57.06	41.49	43.94	43.88	64.96	58.75	61.21	49.47	ND	ND	ND	ND	ND	ND	ND	ND
GCA_000739455.1	*Corynebacterium imitans*	DSM 44264	59.29	ND	ND	ND	ND	34.01	ND	ND	ND	ND	77.15	74.62	ND	77.58	65.78	62.88	54.41	66.92	64.85	ND	ND	ND	ND	ND	ND	ND	ND	ND	ND	ND	53.5	77.2	56.47	42.12	44.99	43.99	67.09	56.5	62.24	49.16	ND	ND	ND	ND	ND	ND	ND	ND
GCA_000577555.1	*Corynebacterium jeddahense*	JCB	ND	ND	ND	30.55	ND	ND	ND	ND	ND	ND	48.01	73.33	ND	76.02	49.63	63.65	55.82	64.66	64.61	33.69	33.45	35.51	31.25	31.52	30.45	31.05	ND	ND	ND	ND	53.43	79.02	56.39	42.29	45.8	44.11	67.39	57.14	61.74	50.56	ND	ND	ND	ND	ND	ND	ND	ND
GCA_000163435.1	*Corynebacterium jeikeium*	ATCC 43734	ND	ND	ND	ND	ND	ND	ND	ND	ND	ND	46.56	71.58	ND	71.46	50	58.74	56.67	58.87	61.42	ND	ND	ND	ND	ND	ND	ND	ND	ND	ND	ND	58.06	72.73	46.39	51.69	33.98	41.96	65.2	61.7	61.18	39.64	ND	ND	ND	ND	ND	ND	ND	ND
GCA_000023145.1	*Corynebacterium kroppenstedtii* *	DSM 44385	ND	ND	ND	ND	ND	32.08	ND	ND	ND	ND	70.32	76.39	ND	ND	83.93	ND	ND	ND	30.65	34.8	34.84	37.31	34.43	37.23	34.31	33.7	35.71	ND	ND	ND	57.69	77.07	49.23	40.65	39.02	43.13	65.79	59.49	57.67	ND	ND	ND	ND	ND	ND	ND	ND	ND
GCA_000980835.1	*Corynebacterium kutscheri*	DSM 20755	61.58	ND	ND	ND	33.65	35.87	ND	ND	ND	ND	84.43	80.57	45.12	81.43	88.43	69.23	71.67	72.93	72.03	ND	ND	ND	ND	ND	ND	ND	ND	ND	ND	ND	53.5	72.96	50	37.65	43.47	41.26	67.98	59.67	68.01	49.87	ND	ND	ND	ND	ND	ND	ND	ND
GCA_001274895.1	*Corynebacterium lactis**	RW2-5	46.58	ND	ND	ND	ND	ND	ND	ND	ND	ND	72.11	81.8	40.3	ND	85.1	ND	ND	ND	30.89	ND	ND	ND	ND	ND	ND	ND	ND	ND	ND	50.91	57.88	75.8	53.33	45.62	45.51	44.78	67.11	60.76	60.17	49.62	ND	ND	ND	ND	ND	ND	ND	ND
GCA_000159635.1	*Corynebacterium lipophiloflavum*	DSM 44291	ND	ND	ND	ND	ND	ND	ND	ND	ND	ND	46.42	72.33	ND	76.22	51.69	62.42	55.67	65.04	62.37	ND	ND	ND	ND	ND	ND	ND	ND	ND	ND	ND	55.63	75.24	56.25	45.77	46.2	43.72	67.69	60.16	61.73	50.14	ND	ND	ND	ND	ND	ND	ND	ND
GCA_001412085.1	*Corynebacterium lowii*	R-50085	ND	ND	ND	ND	ND	ND	ND	ND	ND	ND	47.39	75.73	ND	79.88	54.14	66.85	59.22	71.8	70.58	ND	ND	ND	ND	ND	ND	ND	ND	ND	ND	ND	57.86	76.85	55.39	40.22	45.88	44.52	68.86	63.29	62.69	51.39	ND	ND	ND	ND	ND	ND	ND	ND
GCA_000379425.1	*Corynebacterium lubricantis*	DSM 45231	46.02	ND	ND	ND	ND	33.2	ND	ND	ND	ND	84.53	79.35	33.82	75.39	68.24	64.49	63.24	67.29	64.93	32.13	32.17	35.53	32.23	32.23	31.18	32.49	33.58	ND	ND	ND	55.99	74.45	54.52	41.37	42.82	44.11	67.54	59.07	66.16	50.66	ND	ND	ND	ND	ND	ND	ND	ND
GCA_000835165.1	*Corynebacterium marinum*	DSM 44953	50.99	ND	ND	ND	ND	ND	ND	ND	ND	ND	86.37	79.86	ND	80.43	68.05	70.68	68.51	70.3	71.91	ND	ND	ND	ND	ND	ND	ND	ND	ND	ND	53.56	58.18	78.14	54.27	42.63	50.18	45.18	69.74	58.98	65.26	50.91	ND	ND	ND	ND	ND	ND	ND	ND
GCA_000442645.1	*Corynebacterium maris*	DSM 45190	46.12	ND	ND	ND	ND	32.26	ND	ND	ND	ND	82.69	76.82	41.1	75.92	65.36	66.4	67.56	69.81	68.14	34.31	31.36	34.33	33.98	32.46	33.82	30.8	32.23	ND	ND	ND	54.64	76.18	52.54	41.48	48.21	44.93	66.67	63.25	64	49.33	ND	ND	ND	ND	ND	ND	ND	ND
GCA_000420605.1	*Corynebacterium massiliense*	DSM 45435	43.52	ND	ND	ND	30.91	37.16	ND	ND	ND	ND	60.29	73.55	ND	77.97	57.23	64.56	55.54	66.92	66.41	32.49	33.45	34.41	31.99	31.9	32.62	30.99	34.77	ND	ND	ND	54.3	74.52	53.08	41.27	44.09	48.09	69.3	61.44	61.89	52.39	ND	ND	ND	ND	ND	ND	ND	ND
GCA_000375365.1	*Corynebacterium mastitidis*	DSM 44356	ND	ND	ND	ND	30.37	32.09	ND	ND	ND	ND	49.07	77.05	ND	80.55	56.77	66.48	59.22	69.78	70.75	ND	ND	ND	ND	ND	ND	ND	ND	ND	ND	ND	63.91	79.48	55.21	40.47	47.24	44.66	70.74	59.92	61.77	52.78	ND	ND	ND	ND	ND	ND	ND	ND
GCA_000175375.1	*Corynebacterium matruchotii*	ATCC 14266	50.34	ND	ND	ND	ND	ND	ND	ND	ND	ND	81.32	82.29	48.57	82.17	85.63	67.26	65.1	78.11	70.69	ND	ND	ND	ND	ND	ND	ND	ND	ND	ND	ND	55.93	78.36	51.95	38.79	43.53	41.69	60.53	58.44	67.18	50.89	ND	ND	ND	ND	ND	ND	ND	ND
GCA_000805675.1	*Corynebacterium minutissimum*	ATCC 23348	61.3	ND	ND	ND	ND	ND	ND	ND	ND	ND	80.29	74.62	ND	78.4	68.06	64.46	62.63	65.91	63.64	33.58	32.4	34.8	31.14	33.33	31.77	32.17	31.91	ND	ND	ND	55.27	71.7	51.63	39.18	42.18	44.81	67.37	60.76	66.87	51.07	ND	ND	ND	ND	ND	ND	ND	ND
GCA_001020985.1	*Corynebacterium mustelae*	DSM 45274	50.1	ND	ND	ND	ND	ND	ND	ND	ND	ND	82.6	84.32	49.38	83.14	88.22	69.08	69.66	78.2	68.66	ND	ND	ND	ND	ND	ND	ND	ND	ND	ND	54.74	58.63	77.52	49.4	40.18	45.53	41.92	65.79	60.33	65.81	51.58	ND	ND	ND	ND	ND	ND	ND	ND
GCA_900103625.1	*Corynebacterium mycetoides*	DSM 20632	61.48	ND	ND	ND	ND	35.42	ND	ND	ND	ND	74.36	73.55	ND	76.74	65.38	61.17	53.86	65.41	67.51	ND	ND	ND	ND	ND	ND	ND	ND	ND	ND	ND	61.72	73.97	57.29	43.34	44	42.3	66.23	58.13	65.43	49.33	ND	ND	ND	ND	ND	ND	ND	ND
GCA_000213935.2	*Corynebacterium nuruki*	S6-4	47.35	ND	ND	ND	ND	33.07	ND	ND	ND	ND	48.09	67.55	ND	70.51	55.2	59.68	54.3	56.23	63.09	32.39	30.32	31.99	ND	31.31	30.82	ND	30.46	ND	ND	48.48	57.76	75.16	51.51	48.97	40	39.88	67.54	63.49	60.18	50.68	ND	ND	ND	ND	ND	30.25	ND	34.62
GCA_001412105.1	*Corynebacterium oculi*	R-50187	ND	ND	ND	31.23	ND	32.62	ND	ND	ND	ND	48.12	74.49	ND	80.47	56.58	65.97	59.56	68.42	70.07	ND	ND	ND	ND	ND	ND	ND	ND	ND	ND	ND	61.48	77.88	54.98	39.75	46.34	42.51	70.18	63.98	62.69	51.97	ND	ND	ND	ND	ND	ND	ND	ND
GCA_001941565.1	*Corynebacterium phocae*	M408/89/1	49.06	ND	ND	ND	31.56	ND	ND	ND	ND	ND	77.9	75.84	ND	77.39	67.48	63.64	62.67	66.67	67.57	ND	ND	ND	ND	ND	ND	ND	ND	ND	ND	ND	55.56	76.51	53.41	41.56	45.14	39.39	66.96	61.28	62.2	50	ND	ND	ND	ND	ND	ND	ND	ND
GCA_900092335.1	*Corynebacterium phoceense*	MC1	60.24	ND	ND	ND	ND	34.97	ND	ND	ND	ND	82.76	77.99	ND	78.4	67.82	62.97	61.83	66.92	68.65	ND	ND	ND	ND	ND	ND	ND	ND	ND	ND	ND	55.21	75.16	52.16	40.11	46.26	44.26	70.35	61.18	66.06	48.39	ND	ND	ND	ND	ND	ND	ND	ND
GCA_001044155.1	*Corynebacterium pilosum*	CIP103422	52.59	ND	ND	30.65	30.91	33.61	ND	ND	ND	ND	85.27	77.68	38.24	79.27	68.43	64.18	69.26	66.04	63.89	30.77	31.36	34.07	31.5	30.77	30.69	31.12	33.46	ND	ND	ND	58.39	75.9	55.84	42.78	44.98	42.43	68.42	61.7	61.82	51.44	ND	ND	ND	ND	ND	ND	ND	ND
GCA_000375525.1	*Corynebacterium propinquum*	DSM 44285	58.69	ND	ND	ND	ND	ND	ND	ND	ND	ND	81.58	74.24	ND	79.34	66.92	61.36	60.34	67.67	64.5	ND	ND	ND	ND	ND	ND	ND	ND	ND	ND	ND	53.82	75.64	53.05	40.88	44.48	44.2	68.72	59.57	59.94	49.63	ND	ND	ND	ND	ND	ND	ND	ND
GCA_000688415.1	*Corynebacterium pseudodiphtheriticum*	DSM 44287	57.85	ND	ND	ND	ND	ND	ND	ND	ND	ND	81.95	75.08	ND	78.95	67.11	61.49	60.85	66.92	64.86	ND	ND	ND	ND	ND	ND	ND	ND	ND	ND	ND	54.48	75	52.77	39.79	44.48	43.9	69.64	61.11	60.24	48.89	ND	ND	ND	ND	ND	ND	ND	ND
GCA_002155265.1	*Corynebacterium pseudotuberculosis*	ATCC 19410	50.32	ND	ND	ND	ND	33.98	ND	ND	ND	ND	83.98	86.13	51.43	82.21	86.37	71.51	66.1	76.32	73.59	ND	ND	ND	ND	ND	ND	ND	ND	ND	ND	52.52	59.49	74.53	51.05	40.4	43.64	44.11	66.67	64.56	69.64	51.32	ND	ND	ND	ND	ND	ND	ND	ND
GCA_000411375.1	*Corynebacterium pyruviciproducens*	ATCC BAA-1742	ND	ND	ND	ND	ND	34.33	ND	ND	ND	ND	68.74	77.36	45.88	77.56	81.73	62.21	53.63	74.62	69.46	ND	ND	ND	ND	ND	ND	ND	ND	ND	ND	ND	52.08	80.92	49.22	41.18	46.39	46.3	65.95	63.33	63.47	48.33	ND	ND	ND	ND	ND	ND	ND	ND
GCA_000177535.2	*Corynebacterium resistens*	DSM 45100	ND	ND	ND	ND	ND	ND	ND	ND	ND	ND	47.46	69.43	ND	71.29	50.84	59.05	60.23	57.85	64.98	ND	ND	ND	ND	ND	ND	ND	ND	ND	ND	48.2	57.3	79.02	46.05	41.41	37.67	42.51	68.56	60.71	60.38	47.81	ND	ND	ND	ND	ND	ND	ND	ND
GCA_000833575.1	*Corynebacterium singulare*	IBS B52218	47.38	ND	ND	ND	ND	33.51	ND	ND	ND	ND	79.63	75.97	ND	78.21	67.49	65.75	62.63	65.4	68.58	33.94	32.17	34.43	31.87	32.97	32.13	31.67	31.91	ND	ND	ND	55.4	72.01	52.23	39	42.82	44.08	68.42	60.76	66.57	53.46	ND	ND	ND	ND	ND	ND	ND	ND
GCA_001941505.1	*Corynebacterium sphenisci*	DSM 44792	46.8	ND	ND	ND	32.34	ND	ND	ND	ND	ND	71.32	80.27	ND	68.41	83.58	54.78	53.22	56.98	65.64	ND	ND	ND	ND	ND	ND	ND	ND	ND	ND	54.21	60.65	76.01	ND	46.13	53.1	44.78	62.11	60.24	62.76	51.68	ND	ND	ND	ND	ND	ND	ND	ND
GCA_900113445.1	*Corynebacterium spheniscorum*	PG 39	50	ND	ND	ND	ND	ND	ND	ND	ND	ND	84.15	76.63	ND	80.47	67.35	67.5	65.31	68.77	66.46	ND	ND	ND	ND	ND	ND	ND	ND	ND	ND	50.24	56.6	75.96	49.71	37.96	42.93	41.46	66.23	61.44	67.17	50.89	ND	ND	ND	ND	ND	ND	ND	ND
GCA_000427865.1	*Corynebacterium sputi*	DSM 45148	50.63	ND	ND	ND	ND	32.66	ND	ND	ND	ND	73.63	81.46	ND	66.86	84.82	57.04	53.05	54.34	63.59	ND	ND	ND	30.1	ND	ND	ND	30.16	ND	ND	52.8	54.51	74.29	59.81	44.18	45.91	44.11	58.77	60.17	64.16	51.14	ND	ND	ND	ND	ND	ND	ND	ND
GCA_001941345.1	*Corynebacterium stationis*	622=DSM 20302	60.81	ND	ND	ND	ND	31.82	ND	ND	ND	ND	82.14	77.83	39.08	75.88	68.62	64.58	64.19	67.29	61.31	35.48	31.71	36.26	34.67	34.07	32.49	33.22	31.97	ND	ND	ND	55.56	76.6	52.21	38.65	46.22	42.03	69.16	60.85	65.12	48.91	ND	ND	ND	ND	ND	ND	ND	ND
GCA_000159135.1	*Corynebacterium striatum*	ATCC 6940	59.23	ND	ND	31.29	33.58	35.5	ND	ND	ND	ND	82.69	77.16	47.89	77.82	66.79	66.52	62.61	65.4	65.76	ND	ND	ND	ND	ND	ND	ND	ND	ND	ND	ND	54.9	71.25	53.71	38.79	44.48	43.44	69.64	60.43	65.65	49.21	ND	ND	ND	ND	ND	ND	ND	ND
GCA_000418365.1	*Corynebacterium terpenotabidum*	Y-11	47.31	ND	ND	ND	ND	35.89	ND	ND	ND	ND	46.34	68.88	ND	67.57	47.75	59.82	54.35	56.23	61.5	30	30.65	33.57	ND	30.64	ND	30.07	ND	ND	ND	ND	58.72	73.48	51.03	42.4	39.88	40.75	69.3	57.86	59.94	49.5	ND	ND	ND	ND	ND	ND	ND	ND
GCA_001021045.1	*Corynebacterium testudinoris*	DSM 44614	65.55	ND	ND	ND	ND	ND	ND	ND	ND	ND	87.21	78.51	39.19	80.04	68.13	70.34	68.36	72.18	73.59	ND	ND	ND	ND	ND	ND	ND	ND	ND	ND	51.31	55.75	79.33	52.24	42.71	43.99	45.8	68.53	65.53	68.81	50.78	ND	ND	ND	ND	ND	ND	ND	ND
GCA_900105305.1	*Corynebacterium timonense*	DSM 45434	59.3	ND	ND	31.15	ND	37.1	ND	ND	ND	ND	75.46	73.21	ND	76.02	65.97	63.76	57.34	65.79	63.78	ND	ND	ND	ND	ND	ND	ND	ND	ND	ND	ND	ND	75.48	57.86	44.81	44.17	44.26	66.52	59.75	59.94	49.29	ND	ND	ND	ND	ND	ND	ND	ND
GCA_000372445.1	*Corynebacterium ulceribovis*	DSM 45146	49.36	ND	ND	ND	ND	32.51	ND	ND	ND	ND	45.69	72.01	ND	68.02	51.4	59.55	51.01	57.89	64.52	ND	ND	ND	ND	ND	ND	ND	ND	ND	ND	ND	64.16	76.75	51.69	44.79	40.34	42.67	64.47	56.17	61.92	48.44	ND	ND	ND	ND	ND	ND	ND	ND
GCA_000069945.1	*Corynebacterium urealyticum*	DSM 7109	ND	ND	ND	ND	ND	ND	ND	ND	ND	ND	46.31	69.52	ND	68.54	46.84	57.91	57.57	59.7	64.29	30.29	30.82	31.21	31.27	30.87	ND	ND	30.2	ND	ND	ND	58.8	74.84	43.97	40.84	40.87	42.47	67.54	60.52	61.11	46.62	ND	ND	ND	ND	ND	ND	ND	ND
GCA_000747315.1	*Corynebacterium ureicelerivorans*	IMMIB RIV-2301	ND	ND	ND	ND	ND	ND	ND	ND	ND	ND	48.48	72.84	ND	76.61	50.19	62.88	54.39	63.16	63.95	33.45	33.8	35.51	32.35	32.25	31.14	31.25	ND	ND	ND	ND	56.25	78.69	57.5	42.2	46.34	41.73	67.09	57.92	60.06	49.6	ND	ND	ND	ND	ND	ND	ND	ND
GCA_001021065.1	*Corynebacterium uterequi*	DSM 45634	50.45	ND	ND	ND	ND	ND	ND	ND	ND	ND	47.12	60.17	ND	75.44	50.6	63.65	58.88	68.8	64.75	ND	ND	ND	ND	ND	ND	ND	ND	ND	ND	ND	60.09	70.69	55.45	39.51	47.97	46.49	70.93	59.92	64.63	50.66	ND	ND	ND	ND	ND	ND	ND	ND
GCA_000550805.1	*Corynebacterium vitaeruminis*	DSM 20294	51.09	ND	ND	ND	ND	ND	ND	ND	ND	ND	86.45	86.99	50	81.62	88.04	72.22	71.31	76.32	73.37	ND	ND	ND	ND	ND	ND	ND	ND	ND	ND	51.84	60.28	73.63	54.12	41.79	46.82	43.72	65.2	60.59	63.14	54.18	ND	ND	ND	ND	ND	ND	ND	ND
GCA_001552415.1	*Corynebacterium xerosis*	NBRC 16721	50.21	ND	ND	ND	ND	ND	ND	ND	ND	ND	74.57	81.97	49.32	69.01	85.31	60.26	55.86	55.47	69.01	ND	ND	ND	ND	ND	ND	ND	ND	ND	ND	50.86	59.85	77.67	51.45	48.05	40.96	44.08	69.96	62.98	63.3	53.08	ND	ND	ND	ND	ND	ND	ND	ND
**GCA_000296405.1**	***Turicella otitidis****	**ATCC 51513**	57.87	ND	ND	ND	ND	ND	ND	ND	ND	ND	72.5	65.59	ND	ND	64.42	ND	ND	ND	ND	33.33	33.45	34.78	32.12	32.61	31.41	31.52	32.49	ND	ND	ND	57.4	75.09	56.53	39.34	48.74	46.11	63.16	59.09	55.87	ND	ND	ND	ND	ND	ND	ND	ND	ND
GCA_000226215.2	*Dietzia alimentaria*	72	54.57	55.89	55.82	ND	ND	66.8	ND	ND	ND	56.98	66.1	73.77	38.96	60.12	75.99	49.38	55.38	66.54	53.07	33.33	32.87	35.14	33.46	33.46	33.45	31.14	33.96	ND	ND	64.15	58.74	75.56	56.21	48.78	50	49.17	66.23	67.76	55.21	54.81	ND	ND	35.02	ND	34.07	ND	ND	ND
GCA_000024785.1	*Gordonia bronchialis*	DSM 43247	59.45	63.25	66.67	65.54	61.02	71.24	37.41	53.24	ND	58.36	65.91	75.8	ND	61.9	83.15	52.2	60.74	63.06	52.36	32.98	35.06	33.21	31.92	32.48	32.59	30.4	32.14	56.35	38.93	61.48	62.54	75.39	65.95	63.83	52.56	46.15	66.67	70.45	54.98	59.02	ND	ND	ND	ND	ND	ND	ND	ND
GCA_001653095.1	*Hoyosella altamirensis*	NBRC 109631	58.27	64.8	54.08	60.46	58.64	64.37	42.11	57.45	44.06	63.67	66.04	79.02	ND	60.89	77.64	48.87	59.9	68.8	48.32	31.77	33.1	32.36	32.49	31.27	33.1	31.83	30.59	ND	ND	61.99	67.49	79.75	58.7	65.52	53.42	50.14	70.59	68.83	52.55	ND	ND	ND	36.79	ND	35.14	ND	ND	ND
GCA_001293125.1	*Lawsonella clevelandensis*	X1036	ND	ND	ND	ND	ND	ND	ND	ND	ND	ND	50.75	65.65	ND	58.83	57.39	45	40	51.69	52.96	ND	ND	ND	ND	ND	ND	ND	ND	ND	ND	ND	53.36	75.63	51.37	40.5	45.03	44.68	69.03	57.71	51.18	ND	ND	ND	ND	ND	ND	ND	ND	ND
GCA_001552615.1	*Millisia brevis*	NBRC 105863	60.83	63.58	50.65	67.71	62.94	67.62	40.28	56.03	40.82	61.98	66.29	79.7	ND	63.5	79.32	48.53	59.03	67.19	50.33	31.05	33.45	32.75	32.6	30.4	31.18	ND	30.47	59.32	37.22	58.58	63.5	83.39	60.5	67.27	51.79	49.46	67.84	66.67	53.33	59.42	ND	ND	35.69	ND	ND	ND	ND	ND
GCA_000195955.2	*Mycobacterium tuberculosis*	H37Rv	99.97	99.67	100	99.76	100	100	99.37	100	100	100	100	99.83	100	99.81	100	100	100	100	99.89	100	100	100	100	100	100	100	100	100	100	100	99.66	99.68	99.69	99.82	99.73	100	100	99.66	50.3	100	ND	ND	37.14	ND	37.35	ND	ND	ND
GCA_900114685.1	*Nocardia asteroides*	DSM 43373	62.78	69.44	54.17	68.7	62.44	72.1	52.45	64.96	42.45	58.24	67.16	79.27	ND	64.8	80.91	54.66	60.81	69.43	51.31	35.25	33.45	33.46	34.53	31.14	34.41	31.65	31.88	58.22	40.08	61.88	70.37	86.44	53.25	66.49	56.47	46.34	73.48	69.23	56.31	60.26	ND	ND	35.69	ND	36.36	ND	ND	ND
GCA_001047055.1	*Rhodococcus rhodochrous*	NBRC 16069	63.19	71	56.16	67.09	62.84	69.4	45.89	59.15	42.45	62.08	67.35	73.39	ND	66.02	81.09	50.37	61.8	71.26	52.24	32.49	32.06	33.33	32.72	31.14	33.69	30.07	30.3	56.48	40	64.06	66.32	85.42	65.3	66.07	61.41	53.07	74.77	70.85	60.12	65.79	ND	ND	34.19	ND	33.73	ND	ND	ND
GCA_000092825.1	*Segniliparus rotundus*	DSM 44985	58.1	60.6	ND	64.44	62.68	67.23	ND	52.55	ND	60.38	61.58	71.43	ND	63.17	72.4	51.2	54.46	43.65	48.17	63.12	55.56	54.33	61.27	54.86	59.44	56.12	54.21	55.92	40.5	58.56	53.41	80.53	46.82	62.39	53.58	ND	63.93	65.46	49.49	47.92	ND	ND	34.42	ND	35.76	ND	ND	ND
GCA_001552855.1	*Skermania piniformis*	NBRC 15059	62.45	65.12	56.65	69.47	65.28	70.09	50.36	61.03	42.07	57.51	67.04	70.49	ND	62.55	81.39	49.03	62.89	67.68	52.65	32.16	33.83	32.97	32.56	ND	31.54	30.33	31.63	56.66	37.69	65.83	67.04	86.46	63.23	64.95	60	46.74	72.44	68.94	55.49	60.89	ND	ND	34.53	ND	35.05	ND	ND	ND
GCA_000380645.1	*Smaragdicoccus niigatensis*	DSM 44881	55.35	63.25	52.51	66.83	60.54	70.82	55.71	61.27	41.48	62.69	66.29	79.39	ND	66.67	78.82	46.16	56.65	65.28	51.44	61.62	55.14	58.74	60.56	58.04	60.14	60.65	53.05	59.37	40.33	64.94	63.07	83	60.25	66.61	52.69	46.72	66.96	69.48	55.25	55	ND	ND	35.92	ND	33.94	ND	ND	ND
GCA_000524475.1	*Tomitella biformata*	AHU 1821	60.86	66.89	53.12	61.32	59.9	70.28	44.37	60.84	39.16	63.94	66.6	82.34	ND	63.43	76.55	48.31	59.18	70.41	53.25	35.98	35.96	35.57	31.77	33.2	33.84	32.72	34.38	56.67	39.83	62.62	64.89	83.05	65.53	64.08	57.34	52.45	70.93	66.8	53.33	60.26	ND	ND	34.95	ND	35.17	ND	ND	ND
GCA_000092225.1	*Tsukamurella paurometabola*	DSM 20162	58.08	64.55	49.39	64.49	57.61	69.49	38.1	57.75	ND	57.99	66.35	77.24	ND	58.46	79.77	49.51	58.24	64.93	42.23	61.25	48.45	54.55	60.35	56.99	58.1	55.16	50	62.03	38.52	64.32	61.57	78.43	52.13	67.93	56.4	47.65	66.67	68.83	50.93	ND	ND	ND	34.53	ND	34.49	ND	ND	ND
GCA_001598915.1	*Williamsia muralis*	NBRC 105860	61.07	65.89	63.96	66.18	60.68	71.37	44.29	51.8	40.77	60.45	66.11	73.25	ND	62.7	82.75	53.25	60.66	65.79	51.93	62.85	54.58	58.89	60.07	57.49	59.23	59.86	57.35	58.5	38.93	61.93	65.4	80.46	62.63	61.88	57.18	33.33	69.16	70.33	51.18	58.93	ND	ND	32.75	ND	30.8	ND	ND	ND
GCA_900142365.1	*Pseudonocardia thermophila**	DSM 43832	ND	60.4	43.07	49.36	49.26	64.53	ND	47.06	ND	58.94	61.21	73.86	ND	ND	70	ND	32.24	ND	ND	36.33	33.33	35.71	35.12	34.45	38.28	35.55	32.31	ND	ND	51.44	58.82	83.73	56.41	49.73	50.15	44.21	68.97	67.83	53.75	37.22	ND	ND	33.44	ND	37.39	ND	ND	ND

* Reported as mycolic acid absent species by previous experiments.

† ATCC 6931 is not a type genome. It was used as a reference due to the absence of the type genome of *C. amycolatum* .

Abbreviations

fas, 3-oxoacyl-ACP synthase; fabD, Malonyl CoA-acyl carrier protein transacylase; fabH, 3-oxoacyl-[acyl-carrier-protein] synthase 3; kasA, 3-oxoacyl-[acyl-carrier-protein] synthase 1; kasB, 3-oxoacyl-[acyl-carrier-protein] synthase 2; mabA, 3-oxoacyl-[acyl-carrier-protein] reductase; hadA, (3R)-hydroxyacyl-ACP dehydratase subunit alpha; hadB, (3R)-hydroxyacyl-ACP dehydratase subunit beta; hadC, (3R)-hydroxyacyl-ACP dehydratase subunit gamma; inhA, Enoyl-[acyl-carrier-protein] reductase

accD6, Acetyl-CoA carboxylase beta subunit ; accD4, Acyl-CoA carboxylase subunit beta 1; accD5, Acyl-CoA carboxylase subunit beta 2; accA3, Acyl/Acetyl-CoA carboxylase subunit alpha; accE, Acyl/Acetyl-CoA carboxylase subunit epsilon; pks13, Polyketide synthase; fadD32, Long-chain-fatty-acid--AMP ligase; cmrA, Probable short-chain type dehydrogenase/reductase; mmpL3, Trehalose monomycolate exporter

cmaA1, Cyclopropane mycolic acid synthase 1; cmaA2, Cyclopropane mycolic acid synthase 2; umaA1, Possible mycolic acid synthase; pcaA, Cyclopropane mycolic acid synthase 3; mmaA1, Mycolic acid methyltransferase; mmaA2, Cyclopropane mycolic acid synthase; mmaA3, Methoxy mycolic acid synthase; mmaA4, Hydroxymycolate synthase; desA1, Putative acyl-[acyl-carrier-protein] desaturase; desA2, Putative acyl-[acyl-carrier-protein] desaturase; desA3, NADPH-dependent stearoyl-CoA 9-desaturase

menA, 1,4-dihydroxy-2-naphthoate octaprenyltransferase; menB, 1,4-dihydroxy-2-naphthoyl-CoA synthase; menC, o-succinylbenzoate synthase; menD, 2-succinyl-5-enolpyruvyl-6-hydroxy-3-cyclohexene-1-carboxylate synthase; menE, 2-succinylbenzoate--CoA ligase; menF, Isochorismate synthase; menG, Demethylmenaquinone methyltransferase; uppS, Decaprenyl diphosphate synthase; hepST, Geranylgeranyl pyrophosphate synthase; menJ, Menaquinone reductase

mqnA, Chorismate dehydratase; mqnB, Futalosine hydrolase; mqnC, Cyclic dehypoxanthine futalosine synthase; mqnD, 1,4-dihydroxy-6-naphtoate synthase; mqnE, Aminodeoxyfutalosine synthase; SCO4490, Putative decarboxylase; SCO4491, Putative octaprenyltransferase; ubiX, Flavin prenyltransferase
