# Supplementary material for: Phylogeny Trumps Chemotaxonomy: A Case Study Involving Turicella otitidis
Source: Front Microbiol. 2018 Apr 30;9:834. doi: 10.3389/fmicb.2018.00834 (PMC5936774; doi:10.3389/fmicb.2018.00834)
Supplement: Supplementary file 3 [file Table_3.PDF]

Supplementary Table 3. Gene detected in Corynebacterium genomes associated with the biosynthesis of mycolic acids, and menaquinones.

Assembly Accession	Species name	Strain name	Mycolic acid Biosynthesis																														Menaquinone biosynthesis - classical pathway							Elongation		Saturation	Menaquinone biosynthesis - futalosine pathway							
			fas	fabD	fabH	kasA	kasB	mabA	hadA	hadB	hadC	inhA	accD6	accA3	accE	accD4	accD5	pks13	fadD32	cmrA	mmpL3	cmaA1	cmaA2	umaA1	pcaA	mmaA1	mmaA2	mmaA3	mmaA4	desA1	desA2	desA3	menA	menB	menC	menD	menE	menF	menG	uppS	hepST	menJ	mqnA	mqnB	mqnC	mqnD	mqnE	SCO4490	SCO4491	ubiX
GCA_000159115.1	Corynebacterium accolens	ATCC 49725	ND	ND	ND	110	ND	115	ND	ND	ND	ND	434	834	46.6	800	484	1931	612	370	784	140	150	148	138	144	135	149	145	ND	ND	ND	198	458	265	326	224	245	313	269	410	346	ND	ND	ND	ND	ND	ND	ND	ND
GCA_900156035.1	Corynebacterium afermentans subsp. afermentans	DSM 44280	1719	ND	ND	100	ND	ND	ND	ND	ND	ND	760	816	ND	745	664	1937	581	355	741	132	149	149	131	135	132	129	ND	ND	ND	ND	177	489	306	315	226	231	284	276	381	320	ND	ND	ND	ND	ND	ND	ND	ND
GCA_001941425.1	Corynebacterium ammoniagenes	DSM 20306	1862	ND	ND	ND	ND	111	ND	ND	ND	ND	929	824	60.5	766	753	1918	767	365	729	141	145	160	146	145	139	148	140	ND	ND	ND	233	488	249	284	263	257	320	302	409	346	ND	ND	ND	ND	ND	ND	ND	ND
GCA_000755185.1	Corynebacterium amycolatum *	ATCC 6931†	2431	ND	ND	ND	ND	ND	ND	ND	ND	ND	794	878	ND	ND	937	ND	125	ND	ND	137	144	155	129	134	133	147	136	ND	ND	444	239	485	287	354	205	257	305	283	347	335	ND	ND	ND	ND	ND	ND	ND	ND
GCA_900156665.1	Corynebacterium appendicis	DSM 44531	ND	ND	ND	107	117	ND	ND	ND	ND	ND	427	818	46.6	743	486	1810	635	380	739	132	153	150	139	138	ND	134	141	ND	ND	ND	150	464	241	311	236	255	306	284	348	350	ND	ND	ND	ND	ND	ND	ND	ND
GCA_001941445.1	Corynebacterium aquilae	S-613	2561	ND	ND	ND	114	124	ND	ND	ND	ND	902	880	72	808	981	2048	771	372	907	ND	ND	ND	ND	ND	ND	ND	ND	ND	ND	451	162	495	224	308	206	242	327	293	424	334	ND	ND	ND	ND	ND	ND	ND	ND
GCA_000590555.1	Corynebacterium argentoratense	DSM 44202	2588	ND	ND	ND	ND	ND	ND	ND	ND	ND	906	882	67.4	835	989	2038	770	353	842	ND	ND	ND	ND	ND	ND	ND	ND	ND	ND	441	160	454	236	298	223	233	313	296	401	316	ND	ND	ND	ND	ND	ND	ND	ND
GCA_000732945.1	Corynebacterium atypicum	R2070	1256	ND	ND	ND	ND	ND	ND	ND	ND	ND	453	716	ND	755	528	1899	710	384	854	131	135	159	129	132	123	132	130	ND	ND	ND	235	508	227	315	199	245	318	270	412	318	ND	ND	ND	ND	ND	ND	ND	ND
GCA_000767255.1	Corynebacterium auriscanis	CIP 106629	1419	ND	ND	ND	ND	101	ND	ND	ND	ND	448	842	ND	730	485	1214	657	295	744	ND	ND	ND	ND	ND	ND	ND	ND	ND	ND	365	258	489	214	357	153	231	325	246	344	342	ND	ND	ND	ND	ND	ND	ND	ND
GCA_000183325.2	Corynebacterium bovis	DSM 20582	ND	ND	ND	ND	ND	ND	ND	ND	ND	ND	470	858	40	711	513	615	457	273	734	ND	ND	132	ND	ND	ND	ND	ND	ND	ND	ND	108	485	135	122	144	221	304	238	383	330	ND	ND	ND	ND	ND	ND	ND	ND
GCA_000344785.1	Corynebacterium callunae	DSM 20147	4985	ND	ND	ND	ND	ND	ND	ND	ND	ND	1038	1027	79	953	887	2636	1046	445	1145	137	146	149	140	144	129	140	145	ND	ND	ND	249	481	275	309	233	269	329	286	626	352	ND	ND	ND	ND	ND	ND	ND	ND
GCA_000980815.1	Corynebacterium camporealensis	DSM 44610	2336	ND	ND	112	ND	118	ND	ND	ND	ND	855	810	59.7	759	714	1918	745	338	784	ND	ND	ND	ND	ND	ND	ND	ND	ND	ND	ND	230	461	256	322	210	273	300	287	384	346	ND	ND	ND	ND	ND	ND	ND	ND
GCA_000372085.1	Corynebacterium capitovis	DSM 44611	3308	ND	ND	110	ND	103	ND	ND	ND	ND	803	810	46.2	775	702	1893	617	372	787	ND	ND	ND	ND	ND	ND	ND	ND	ND	ND	ND	147	453	248	323	240	231	313	267	405	311	ND	ND	ND	ND	ND	ND	ND	ND
GCA_000550785.1	Corynebacterium casei	LMG S-19264	2034	ND	ND	ND	ND	ND	ND	ND	ND	ND	921	803	56.6	753	751	1996	782	364	764	ND	ND	ND	ND	ND	ND	ND	ND	ND	ND	ND	180	493	299	279	252	257	320	287	380	324	ND	ND	ND	ND	ND	ND	ND	ND
GCA_000379705.1	Corynebacterium caspium*	DSM 44850	1890	ND	ND	ND	118	107	ND	ND	ND	ND	791	830	ND	770	689	1979	636	345	895	ND	ND	ND	ND	ND	ND	ND	ND	ND	ND	ND	240	499	280	298	219	270	305	300	416	351	ND	ND	ND	ND	ND	ND	ND	ND
GCA_000372385.1	Corynebacterium ciconiae*	DSM 44920	2058	ND	ND	ND	ND	103	ND	ND	ND	ND	848	872	73.9	798	971	1939	772	383	862	ND	ND	ND	ND	ND	ND	ND	ND	ND	ND	403	149	459	291	295	227	259	327	273	379	364	ND	ND	ND	ND	ND	ND	ND	ND
GCA_900105505.1	Corynebacterium coyleae	DSM 44184	1744	ND	ND	ND	ND	109	ND	ND	ND	ND	773	864	ND	776	678	1877	608	375	769	ND	ND	ND	ND	ND	ND	ND	ND	ND	ND	ND	214	487	302	313	227	246	303	276	382	322	ND	ND	ND	ND	ND	ND	ND	ND
GCA_001643015.1	Corynebacterium crudilactis	JZ16	5220	ND	ND	ND	ND	116	ND	ND	ND	ND	1092	1033	112	986	1029	2913	1120	476	1222	ND	ND	ND	ND	ND	ND	ND	ND	ND	ND	ND	207	471	283	318	221	264	324	290	620	372	ND	ND	ND	ND	ND	ND	ND	ND
GCA_900111265.1	Corynebacterium cystitidis	DSM 20524	3410	ND	ND	ND	124	116	ND	ND	ND	ND	951	837	51.6	777	770	1884	728	338	723	101	ND	102	ND	ND	106	100	102	ND	ND	ND	184	484	278	304	213	238	320	283	434	320	ND	ND	ND	ND	ND	ND	ND	ND
GCA_001277995.1	Corynebacterium deserti	GIMN1.010	5195	ND	ND	ND	ND	179	ND	ND	ND	ND	1067	1041	97.4	974	937	2806	1056	473	1228	136	154	157	139	150	129	143	147	ND	ND	ND	177	481	306	319	225	268	328	317	633	361	ND	ND	ND	ND	ND	ND	ND	ND
GCA_001457455.1	Corynebacterium diphtheriae	NCTC 11397	2557	ND	ND	ND	ND	135	ND	ND	ND	ND	927	962	68.9	836	987	2104	764	411	873	ND	ND	ND	ND	ND	ND	ND	ND	ND	ND	436	201	474	278	195	214	245	303	278	432	336	ND	ND	ND	ND	ND	ND	ND	ND
GCA_000767055.1	Corynebacterium doosanense	CAU 212	3411	ND	ND	ND	ND	ND	ND	ND	ND	ND	902	884	ND	767	731	1994	797	390	720	ND	ND	ND	ND	ND	ND	ND	ND	ND	ND	ND	218	494	249	310	221	258	295	284	380	338	ND	ND	ND	ND	ND	ND	ND	ND
GCA_000011305.1	Corynebacterium efficiens	YS-314	4791	ND	ND	ND	ND	134	ND	ND	ND	ND	1088	1019	86.3	916	1005	2092	1048	422	1080	ND	ND	ND	ND	ND	ND	ND	ND	ND	ND	ND	232	486	281	333	237	260	328	228	569	356	ND	ND	ND	ND	ND	ND	ND	ND
GCA_001021025.1	Corynebacterium epidermidicanis	DSM 45586	2636	ND	ND	ND	ND	104	ND	ND	ND	ND	867	924	70.1	819	985	2162	764	405	887	ND	ND	ND	ND	ND	ND	ND	ND	ND	ND	433	183	499	269	350	228	232	295	290	401	349	ND	ND	ND	ND	ND	ND	ND	ND
GCA_000525655.1	Corynebacterium falsenii	DSM 44353	1308	ND	ND	ND	ND	ND	ND	ND	ND	ND	450	848	ND	726	491	1308	687	301	801	ND	ND	ND	ND	ND	ND	ND	ND	ND	ND	373	252	486	187	324	165	234	323	254	380	331	ND	ND	ND	ND	ND	ND	ND	ND
GCA_001941465.1	Corynebacterium flavescens	OJ8	1924	ND	ND	ND	116	111	ND	ND	ND	ND	865	853	56.2	701	696	1932	723	354	817	ND	ND	ND	ND	ND	ND	ND	ND	ND	ND	ND	150	473	266	323	237	248	322	296	397	340	ND	ND	ND	ND	ND	ND	ND	ND
GCA_900176865.1	Corynebacterium fournierii	Marseille-P2948	ND	ND	ND	ND	ND	101	ND	ND	ND	ND	444	837	ND	731	473	801	548	358	753	133	146	150	132	135	132	130	ND	ND	ND	ND	147	490	296	228	229	234	306	273	370	340	ND	ND	ND	ND	ND	ND	ND	ND
GCA_001941485.1	Corynebacterium frankenforstense	ST18	1853	ND	ND	ND	ND	125	ND	ND	ND	ND	775	877	ND	777	693	1914	776	400	865	124	132	144	132	128	125	133	134	ND	ND	ND	241	473	158	256	215	190	336	273	422	305	ND	ND	ND	ND	ND	ND	ND	ND
GCA_000428805.1	Corynebacterium freiburgense	DSM 45254	2553	ND	ND	ND	ND	102	ND	ND	ND	ND	900	899	73.6	780	1007	2048	792	387	783	ND	ND	ND	ND	ND	ND	ND	ND	ND	ND	444	216	491	247	284	211	265	317	278	432	369	ND	ND	ND	ND	ND	ND	ND	ND
GCA_000143825.1	Corynebacterium genitalium	ATCC 33030	ND	ND	ND	ND	122	103	ND	ND	ND	ND	409	786	47.8	707	472	1777	643	374	743	131	146	146	132	129	ND	ND	137	ND	ND	ND	120	455	229	308	224	227	270	295	389	354	ND	ND	ND	ND	ND	ND	ND	ND
GCA_000159595.1	Corynebacterium glucuronolyticum	ATCC 51866	1281	ND	ND	ND	ND	ND	ND	ND	ND	ND	719	898	71.2	794	889	1773	610	331	804	ND	ND	ND	ND	ND	ND	ND	ND	ND	ND	ND	226	518	245	322	239	271	310	305	408	310	ND	ND	ND	ND	ND	ND	ND	ND
GCA_000011325.1	Corynebacterium glutamicum	ATCC 13032	5493	ND	ND	ND	ND	115	ND	ND	ND	ND	1084	1083	146	1013	1098	3122	1170	511	1313	129	145	152	132	144	ND	135	141	ND	ND	ND	234	477	271	320	216	271	320	263	692	363	ND	ND	ND	ND	ND	ND	ND	ND
GCA_000626675.1	Corynebacterium glyciniphilum	AJ 3170	2359	ND	ND	ND	ND	184	ND	ND	ND	ND	478	797	ND	702	548	1163	652	295	761	140	131	144	142	137	128	137	135	ND	ND	394	112	472	229	350	155	204	321	293	370	325	ND	ND	ND	ND	ND	ND	ND	ND
GCA_000341345.1	Corynebacterium halotolerans	YIM 70093	2608	ND	ND	ND	ND	122	ND	ND	ND	ND	983	879	61.2	815	765	1579	811	400	876	125	140	148	126	128	123	127	134	ND	ND	422	167	476	266	342	226	260	330	244	441	343	ND	ND	ND	ND	ND	ND	ND	ND
GCA_000819445.1	Corynebacterium humireducens	DSM 45392	2608	ND	ND	ND	ND	127	ND	ND	ND	ND	977	880	ND	819	766	2123	833	384	887	ND	ND	ND	ND	ND	ND	ND	ND	ND	ND	409	201	473	235	326	215	265	302	259	412	315	ND	ND	ND	ND	ND	ND	ND	ND
GCA_000403725.1	Corynebacterium ihumii	GD7	1699	ND	ND	ND	ND	ND	ND	ND	ND	ND	764	840	ND	747	663	1915	582	356	742	ND	ND	ND	ND	ND	ND	ND	ND	ND	ND	ND	138	488	171	309	223	237	298	276	377	318	ND	ND	ND	ND	ND	ND	ND	ND
GCA_000739455.1	Corynebacterium imitans	DSM 44264	3218	ND	ND	ND	ND	100	ND	ND	ND	ND	822	821	ND	749	696	1854	616	368	784	ND	ND	ND	ND	ND	ND	ND	ND	ND	ND	ND	100	453	251	309	239	228	296	287	376	304	ND	ND	ND	ND	ND	ND	ND	ND
GCA_000577555.1	Corynebacterium jeddahense	JCB	ND	ND	ND	102	ND	ND	ND	ND	ND	ND	464	850	ND	759	490	1880	578	356	743	131	147	154	129	134	131	133	ND	ND	ND	ND	215	491	299	291	235	238	307	280	375	308	ND	ND	ND	ND	ND	ND	ND	ND
GCA_000163435.1	Corynebacterium jeikeium	ATCC 43734	ND	ND	ND	ND	ND	ND	ND	ND	ND	ND	395	757	ND	671	440	1793	637	315	743	ND	ND	ND	ND	ND	ND	ND	ND	ND	ND	ND	166	473	210	165	160	224	306	288	363	288	ND	ND	ND	ND	ND	ND	ND	ND
GCA_000023145.1	Corynebacterium kroppenstedtii *	DSM 44385	ND	ND	ND	ND	ND	114	ND	ND	ND	ND	758	833	ND	ND	905	ND	ND	ND	110	149	149	162	150	162	152	157	163	ND	ND	ND	166	494	259	301	202	258	303	294	357	ND	ND	ND	ND	ND	ND	ND	ND	ND
GCA_000980835.1	Corynebacterium kutscheri	DSM 20755	3269	ND	ND	ND	104	118	ND	ND	ND	ND	951	902	72.4	850	991	2132	845	416	800	ND	ND	ND	ND	ND	ND	ND	ND	ND	ND	ND	264	479	271	302	228	246	305	301	431	377	ND	ND	ND	ND	ND	ND	ND	ND
GCA_001274895.1	Corynebacterium lactis*	RW2-5	1250	ND	ND	ND	ND	ND	ND	ND	ND	ND	795	899	48.1	ND	946	ND	ND	ND	104	ND	ND	ND	ND	ND	ND	ND	ND	ND	ND	443	239	488	266	356	217	254	313	277	349	357	ND	ND	ND	ND	ND	ND	ND	ND
GCA_000159635.1	Corynebacterium lipophiloflavum	DSM 44291	ND	ND	ND	ND	ND	ND	ND	ND	ND	ND	464	833	ND	750	529	1789	618	361	753	ND	ND	ND	ND	ND	ND	ND	ND	ND	ND	ND	214	424	274	227	224	254	290	293	378	319	ND	ND	ND	ND	ND	ND	ND	ND
GCA_001412085.1	Corynebacterium lowii	R-50085	ND	ND	ND	ND	ND	ND	ND	ND	ND	ND	460	877	ND	782	540	1995	694	389	838	ND	ND	ND	ND	ND	ND	ND	ND	ND	ND	ND	248	487	290	282	258	177	309	302	392	333	ND	ND	ND	ND	ND	ND	ND	ND
GCA_000379425.1	Corynebacterium lubricantis	DSM 45231	2144	ND	ND	ND	ND	129	ND	ND	ND	ND	945	845	42.7	768	764	1972	745	358	778	136	141	155	136	138	132	142	139	ND	ND	ND	214	478	271	297	226	251	318	290	410	358	ND	ND	ND	ND	ND	ND	ND	ND
GCA_000835165.1	Corynebacterium marinum	DSM 44953	2597	ND	ND	ND	ND	ND	ND	ND	ND	ND	980	844	ND	818	762	2180	808	389	976	ND	ND	ND	ND	ND	ND	ND	ND	ND	ND	424	214	501	190	328	194	263	308	244	401	350	ND	ND	ND	ND	ND	ND	ND	ND
GCA_000442645.1	Corynebacterium maris	DSM 45190	2043	ND	ND	ND	ND	104	ND	ND	ND	ND	901	858	57	738	717	1931	809	390	825	135	125	140	131	132	132	129	125	ND	ND	ND	193	464	265	291	233	247	317	293	382	327	ND	ND	ND	ND	ND	ND	ND	ND
GCA_000420605.1	Corynebacterium massiliense	DSM 45435	1781	ND	ND	ND	117	115	ND	ND	ND	ND	574	771	ND	728	536	1836	619	372	811	130	138	145	129	133	132	130	144	ND	ND	ND	239	447	259	309	219	259	317	294	378	334	ND	ND	ND	ND	ND	ND	ND	ND
GCA_000375365.1	Corynebacterium mastitidis	DSM 44356	ND	ND	ND	ND	115	101	ND	ND	ND	ND	463	867	ND	774	555	1971	706	372	840	ND	ND	ND	ND	ND	ND	ND	ND	ND	ND	ND	182	501	267	242	248	229	313	270	388	337	ND	ND	ND	ND	ND	ND	ND	ND
GCA_000175375.1	Corynebacterium matruchotii	ATCC 14266	2611	ND	ND	ND	ND	ND	ND	ND	ND	ND	878	852	56.2	837	971	2063	771	405	457	ND	ND	ND	ND	ND	ND	ND	ND	ND	ND	ND	205	498	237	264	187	213	276	289	420	368	ND	ND	ND	ND	ND	ND	ND	ND
GCA_000805675.1	Corynebacterium minutissimum	ATCC 23348	3157	ND	ND	ND	ND	ND	ND	ND	ND	ND	885	787	ND	790	746	1964	734	360	782	131	139	144	126	135	128	134	132	ND	ND	ND	144	466	285	303	217	253	317	279	401	349	ND	ND	ND	ND	ND	ND	ND	ND
GCA_001020985.1	Corynebacterium mustelae	DSM 45274	2550	ND	ND	ND	ND	ND	ND	ND	ND	ND	927	880	75.1	867	992	2132	822	412	840	ND	ND	ND	ND	ND	ND	ND	ND	ND	ND	447	259	491	264	272	179	235	318	295	412	370	ND	ND	ND	ND	ND	ND	ND	ND
GCA_900103625.1	Corynebacterium mycetoides	DSM 20632	3268	ND	ND	ND	ND	114	ND	ND	ND	ND	824	848	ND	776	729	1829	603	357	750	ND	ND	ND	ND	ND	ND	ND	ND	ND	ND	ND	123	463	257	319	221	250	301	278	374	320	ND	ND	ND	ND	ND	ND	ND	ND
GCA_000213935.2	Corynebacterium nuruki	S6-4	2224	ND	ND	ND	ND	101	ND	ND	ND	ND	436	764	ND	714	491	1701	608	275	707	123	125	144	ND	132	114	ND	121	ND	ND	363	145	482	218	207	123	201	312	268	373	235	ND	ND	ND	ND	ND	143	ND	100
GCA_001412105.1	Corynebacterium oculi	R-50187	ND	ND	ND	105	ND	101	ND	ND	ND	ND	482	845	ND	760	584	2040	720	379	798	ND	ND	ND	ND	ND	ND	ND	ND	ND	ND	ND	218	493	283	239	245	248	326	301	384	343	ND	ND	ND	ND	ND	ND	ND	ND
GCA_001941565.1	Corynebacterium phocae	M408/89/1	2330	ND	ND	ND	104	ND	ND	ND	ND	ND	865	827	ND	741	749	1863	739	330	764	ND	ND	ND	ND	ND	ND	ND	ND	ND	ND	ND	216	470	271	298	215	208	313	293	389	336	ND	ND	ND	ND	ND	ND	ND	ND
GCA_900092335.1	Corynebacterium phoceense	MC1	2138	ND	ND	ND	ND	108	ND	ND	ND	ND	895	785	ND	768	739	1845	706	350	776	ND	ND	ND	ND	ND	ND	ND	ND	ND	ND	ND	180	481	268	314	226	261	328	286	385	329	ND	ND	ND	ND	ND	ND	ND	ND
GCA_001044155.1	Corynebacterium pilosum	CIP103422	1419	ND	ND	110	123	116	ND	ND	ND	ND	951	899	50.8	686	765	1908	408	362	768	123	131	144	131	127	124	132	133	ND	ND	ND	170	486	280	337	166	253	323	291	267	344	ND	ND	ND	ND	ND	ND	ND	ND
GCA_000375525.1	Corynebacterium propinquum	DSM 44285	1829	ND	ND	ND	ND	ND	ND	ND	ND	ND	883	823	ND	784	731	1860	721	368	819	ND	ND	ND	ND	ND	ND	ND	ND	ND	ND	ND	233	480	232	318	219	267	322	283	400	362	ND	ND	ND	ND	ND	ND	ND	ND
GCA_000688415.1	Corynebacterium pseudodiphtheriticum	DSM 44287	1808	ND	ND	ND	ND	ND	ND	ND	ND	ND	907	816	ND	781	747	1867	729	370	820	ND	ND	ND	ND	ND	ND	ND	ND	ND	ND	ND	233	477	228	315	219	269	325	291	398	364	ND	ND	ND	ND	ND	ND	ND	ND
GCA_002155265.1	Corynebacterium pseudotuberculosis	ATCC 19410	2634	ND	ND	ND	ND	129	ND	ND	ND	ND	950	920	69.3	833	962	2197	786	419	875	ND	ND	ND	ND	ND	ND	ND	ND	ND	ND	449	211	480	252	313	221	241	314	313	453	340	ND	ND	ND	ND	ND	ND	ND	ND
GCA_000411375.1	Corynebacterium pyruviciproducens	ATCC BAA-1742	ND	ND	ND	ND	ND	124	ND	ND	ND	ND	759	897	73.2	784	924	1882	568	375	865	ND	ND	ND	ND	ND	ND	ND	ND	ND	ND	ND	236	516	235	315	228	260	318	281	398	298	ND	ND	ND	ND	ND	ND	ND	ND
GCA_000177535.2	Corynebacterium resistens	DSM 45100	ND	ND	ND	ND	ND	ND	ND	ND	ND	ND	456	808	ND	743	506	1845	652	294	720	ND	ND	ND	ND	ND	ND	ND	ND	ND	ND	220	191	488	216	345	155	235	308	248	339	330	ND	ND	ND	ND	ND	ND	ND	ND
GCA_000833575.1	Corynebacterium singulare	IBS B52218	2236	ND	ND	ND	ND	103	ND	ND	ND	ND	886	826	ND	788	743	1450	729	356	800	133	138	142	125	134	130	133	130	ND	ND	ND	178	466	261	311	220	253	316	275	370	341	ND	ND	ND	ND	ND	ND	ND	ND
GCA_001941505.1	Corynebacterium sphenisci	DSM 44792	672	ND	ND	ND	104	ND	ND	ND	ND	ND	781	810	ND	681	929	1394	538	271	705	ND	ND	ND	ND	ND	ND	ND	ND	ND	ND	440	105	424	ND	274	147	240	245	267	315	334	ND	ND	ND	ND	ND	ND	ND	ND
GCA_900113445.1	Corynebacterium spheniscorum	PG 39	2500	ND	ND	ND	ND	ND	ND	ND	ND	ND	916	805	ND	818	769	2049	784	370	837	ND	ND	ND	ND	ND	ND	ND	ND	ND	ND	436	248	488	283	252	240	226	301	275	405	356	ND	ND	ND	ND	ND	ND	ND	ND
GCA_000427865.1	Corynebacterium sputi	DSM 45148	2468	ND	ND	ND	ND	100	ND	ND	ND	ND	797	914	ND	710	921	1607	608	245	774	ND	ND	ND	110	ND	ND	ND	122	ND	ND	444	182	480	278	399	248	258	263	280	386	357	ND	ND	ND	ND	ND	ND	ND	ND
GCA_001941345.1	Corynebacterium stationis	622=DSM 20302	1907	ND	ND	ND	ND	101	ND	ND	ND	ND	892	798	57.4	795	735	1946	765	364	729	150	147	163	152	150	139	147	134	ND	ND	ND	145	486	275	291	262	247	323	244	389	347	ND	ND	ND	ND	ND	ND	ND	ND
GCA_000159135.1	Corynebacterium striatum	ATCC 6940	1824	ND	ND	105	112	119	ND	ND	ND	ND	908	847	62.4	779	736	1971	723	348	777	ND	ND	ND	ND	ND	ND	ND	ND	ND	ND	ND	167	476	259	302	229	257	320	259	404	357	ND	ND	ND	ND	ND	ND	ND	ND
GCA_000418365.1	Corynebacterium terpenotabidum	Y-11	2317	ND	ND	ND	ND	119	ND	ND	ND	ND	466	729	ND	711	499	1724	587	290	686	121	132	140	ND	124	ND	125	ND	ND	ND	ND	185	467	228	224	154	190	311	273	363	330	ND	ND	ND	ND	ND	ND	ND	ND
GCA_001021045.1	Corynebacterium testudinoris	DSM 44614	3554	ND	ND	ND	ND	ND	ND	ND	ND	ND	943	837	56.6	782	753	2180	818	394	909	ND	ND	ND	ND	ND	ND	ND	ND	ND	ND	430	215	491	241	342	226	233	312	256	404	324	ND	ND	ND	ND	ND	ND	ND	ND
GCA_900105305.1	Corynebacterium timonense	DSM 45434	1816	ND	ND	103	ND	104	ND	ND	ND	ND	818	835	ND	755	716	1810	639	361	770	ND	ND	ND	ND	ND	ND	ND	ND	ND	ND	ND	ND	443	287	336	229	229	303	277	397	296	ND	ND	ND	ND	ND	ND	ND	ND
GCA_000372445.1	Corynebacterium ulceribovis	DSM 45146	1359	ND	ND	ND	ND	102	ND	ND	ND	ND	444	802	ND	637	477	1793	615	302	731	ND	ND	ND	ND	ND	ND	ND	ND	ND	ND	ND	256	489	222	332	162	214	283	268	378	335	ND	ND	ND	ND	ND	ND	ND	ND
GCA_000069945.1	Corynebacterium urealyticum	DSM 7109	ND	ND	ND	ND	ND	ND	ND	ND	ND	ND	444	754	ND	726	482	1714	671	303	783	115	128	133	134	127	ND	ND	124	ND	ND	ND	228	474	197	327	164	215	305	284	379	336	ND	ND	ND	ND	ND	ND	ND	ND
GCA_000747315.1	Corynebacterium ureicelerivorans	IMMIB RIV-2301	ND	ND	ND	ND	ND	ND	ND	ND	ND	ND	466	827	ND	754	489	1867	574	349	786	133	150	152	133	137	134	131	ND	ND	ND	ND	184	494	302	312	232	238	305	268	355	318	ND	ND	ND	ND	ND	ND	ND	ND
GCA_001021065.1	Corynebacterium uterequi	DSM 45634	2443	ND	ND	ND	ND	ND	ND	ND	ND	ND	454	671	ND	738	486	1791	613	373	758	ND	ND	ND	ND	ND	ND	ND	ND	ND	ND	ND	160	460	243	302	253	271	326	264	392	361	ND	ND	ND	ND	ND	ND	ND	ND
GCA_000550805.1	Corynebacterium vitaeruminis	DSM 20294	2579	ND	ND	ND	ND	ND	ND	ND	ND	ND	951	904	53.5	848	988	2281	849	426	864	ND	ND	ND	ND	ND	ND	ND	ND	ND	ND	418	224	466	249	308	226	244	300	294	446	369	ND	ND	ND	ND	ND	ND	ND	ND
GCA_001552415.1	Corynebacterium xerosis	NBRC 16721	2368	ND	ND	ND	ND	ND	ND	ND	ND	ND	800	871	64.7	719	950	1219	653	289	792	ND	ND	ND	ND	ND	ND	ND	ND	ND	ND	418	154	468	244	287	177	246	313	254	363	380	ND	ND	ND	ND	ND	ND	ND	ND
**GCA_000296405.1**	**Turicella otitidis***	**ATCC 51513**	1685	ND	ND	ND	ND	ND	ND	ND	ND	ND	755	699	ND	ND	673	ND	ND	ND	ND	130	143	142	128	133	127	134	140	ND	ND	ND	177	446	195	243	231	204	287	237	345	ND	ND	ND	ND	ND	ND	ND	ND	ND
GCA_000226215.2	Dietzia alimentaria	72	1628	301	323	ND	ND	327	ND	ND	ND	305	735	828	50.8	644	842	1335	652	350	574	131	143	151	131	137	138	129	140	ND	ND	533	207	484	221	304	207	271	288	314	308	394	ND	ND	183	ND	186	ND	ND	ND
GCA_000024785.1	Gordonia bronchialis	DSM 43247	1866	323	424	513	479	340	104	154	ND	303	717	788	ND	664	886	1638	717	331	681	130	128	135	116	127	124	122	122	392	185	551	214	488	234	565	277	263	295	318	326	435	ND	ND	ND	ND	ND	ND	ND	ND
GCA_001653095.1	Hoyosella altamirensis	NBRC 109631	3179	276	354	420	427	322	124	172	129	321	726	833	ND	644	879	1387	716	368	595	137	141	142	144	143	139	144	147	ND	ND	514	199	519	303	639	305	284	331	353	331	ND	ND	ND	196	ND	186	ND	ND	ND
GCA_001293125.1	Lawsonella clevelandensis	X1036	ND	ND	ND	ND	ND	ND	ND	ND	ND	ND	499	696	ND	607	561	1275	423	253	560	ND	ND	ND	ND	ND	ND	ND	ND	ND	ND	ND	220	488	257	305	217	236	318	291	318	ND	ND	ND	ND	ND	ND	ND	ND	ND
GCA_001552615.1	Millisia brevis	NBRC 105863	1720	338	311	526	480	315	103	161	106	305	727	815	ND	650	853	993	691	341	623	124	139	140	130	128	125	ND	119	398	181	463	190	514	251	654	251	310	301	331	328	405	ND	ND	118	ND	ND	ND	ND	ND
GCA_000195955.2	Mycobacterium tuberculosis	H37Rv	5635	481	679	816	845	481	322	288	345	547	927	1115	354	1009	1091	3249	1304	497	1619	573	637	598	572	592	569	615	600	705	548	855	406	646	545	1011	628	629	473	608	285	677	ND	ND	197	ND	197	ND	ND	ND
GCA_900114685.1	Nocardia asteroides	DSM 43373	3328	275	347	542	519	335	145	185	125	294	729	828	ND	691	870	1272	734	353	644	141	136	134	133	126	138	132	130	377	176	529	278	538	217	619	333	253	340	302	336	416	ND	ND	193	ND	191	ND	ND	ND
GCA_001047055.1	Rhodococcus rhodochrous	NBRC 16069	3273	299	347	508	496	334	133	176	109	339	760	800	ND	691	900	1083	741	371	592	138	138	142	136	131	141	135	125	380	178	557	262	530	298	659	351	301	332	322	345	448	ND	ND	196	ND	182	ND	ND	ND
GCA_000092825.1	Segniliparus rotundus	DSM 44985	3125	315	ND	525	502	313	ND	152	ND	281	686	738	ND	644	787	1265	637	155	582	340	305	323	332	306	330	303	291	362	169	523	161	498	195	549	224	ND	271	305	211	320	ND	ND	185	ND	185	ND	ND	ND
GCA_001552855.1	Skermania piniformis	NBRC 15059	3105	321	351	539	516	283	129	176	115	300	747	744	ND	597	887	637	722	300	593	111	101	110	100	ND	105	114	115	381	176	514	269	512	273	523	262	225	272	299	313	421	ND	ND	192	ND	180	ND	ND	ND
GCA_000380645.1	Smaragdicoccus niigatensis	DSM 44881	2829	257	323	533	483	342	145	185	112	325	740	838	ND	714	875	1315	652	352	661	367	320	359	356	358	357	356	315	405	181	506	245	515	280	685	310	230	307	348	353	419	ND	ND	195	ND	180	ND	ND	ND
GCA_000524475.1	Tomitella biformata	AHU 1821	3289	277	325	491	478	352	131	174	109	320	711	687	ND	610	835	1410	706	373	705	153	155	159	139	147	145	153	154	383	162	534	249	510	269	599	298	286	327	317	303	457	ND	ND	191	ND	185	ND	ND	ND
GCA_000092225.1	Tsukamurella paurometabola	DSM 20162	2987	293	298	493	456	337	107	177	ND	298	734	816	ND	631	872	1558	718	354	525	322	252	306	311	319	303	285	280	413	172	542	233	491	200	611	319	261	304	346	278	ND	ND	ND	195	ND	196	ND	ND	ND
GCA_001598915.1	Williamsia muralis	NBRC 105860	3248	291	390	489	444	348	118	153	103	318	728	851	ND	665	882	1753	700	349	666	374	319	349	360	349	355	353	324	376	177	568	223	509	317	550	316	102	312	324	295	372	ND	ND	129	ND	103	ND	ND	ND
GCA_900142365.1	Pseudonocardia thermophila*	DSM 43832	ND	265	249	358	362	300	ND	122	ND	327	624	770	ND	ND	714	ND	171	ND	ND	150	150	151	143	139	164	152	129	ND	ND	357	231	508	225	295	167	132	294	284	285	135	ND	ND	170	ND	177	ND	ND	ND

* Reported as mycolic acid absent species by previous experiments.

† ATCC 6931 is not a type genome. It was used as a reference due to the absence of the type genome of *C. amycolatum* .

Abbreviations

fas, 3-oxoacyl-ACP synthase; fabD, Malonyl CoA-acyl carrier protein transacylase; fabH, 3-oxoacyl-[acyl-carrier-protein] synthase 3; kasA, 3-oxoacyl-[acyl-carrier-protein] synthase 1; kasB, 3-oxoacyl-[acyl-carrier-protein] synthase 2; mabA, 3-oxoacyl-[acyl-carrier-protein] reductase; hadA, (3R)-hydroxyacyl-ACP dehydratase subunit alpha; hadB, (3R)-hydroxyacyl-ACP dehydratase subunit beta; hadC, (3R)-hydroxyacyl-ACP dehydratase subunit gamma; inhA, Enoyl-[acyl-carrier-protein] reductase

accD6, Acetyl-CoA carboxylase beta subunit ; accD4, Acyl-CoA carboxylase subunit beta 1; accD5, Acyl-CoA carboxylase subunit beta 2; accA3, Acyl/Acetyl-CoA carboxylase subunit alpha; accE, Acyl/Acetyl-CoA carboxylase subunit epsilon; pks13, Polyketide synthase; fadD32, Long-chain-fatty-acid--AMP ligase; cmrA, Probable short-chain type dehydrogenase/reductase; mmpL3, Trehalose monomycolate exporter

cmaA1, Cyclopropane mycolic acid synthase 1; cmaA2, Cyclopropane mycolic acid synthase 2; umaA1, Possible mycolic acid synthase; pcaA, Cyclopropane mycolic acid synthase 3; mmaA1, Mycolic acid methyltransferase; mmaA2, Cyclopropane mycolic acid synthase; mmaA3, Methoxy mycolic acid synthase; mmaA4, Hydroxymycolate synthase; desA1, Putative acyl-[acyl-carrier-protein] desaturase; desA2, Putative acyl-[acyl-carrier-protein] desaturase; desA3, NADPH-dependent stearoyl-CoA 9-desaturase

menA, 1,4-dihydroxy-2-naphthoate octaprenyltransferase; menB, 1,4-dihydroxy-2-naphthoyl-CoA synthase; menC, o-succinylbenzoate synthase; menD, 2-succinyl-5-enolpyruvyl-6-hydroxy-3-cyclohexene-1-carboxylate synthase; menE, 2-succinylbenzoate--CoA ligase; menF, Isochorismate synthase; menG, Demethylmenaquinone methyltransferase; uppS, Decaprenyl diphosphate synthase; hepST, Geranylgeranyl pyrophosphate synthase; menJ, Menaquinone reductase

mqnA, Chorismate dehydratase; mqnB, Futalosine hydrolase; mqnC, Cyclic dehypoxanthine futalosine synthase; mqnD, 1,4-dihydroxy-6-naphtoate synthase; mqnE, Aminodeoxyfutalosine synthase; SCO4490, Putative decarboxylase; SCO4491, Putative octaprenyltransferase; ubiX, Flavin prenyltransferase
