# Supplementary material for: Phylogeny Trumps Chemotaxonomy: A Case Study Involving Turicella otitidis
Source: Front Microbiol. 2018 Apr 30;9:834. doi: 10.3389/fmicb.2018.00834 (PMC5936774; doi:10.3389/fmicb.2018.00834)
Supplement: Supplementary file 4 [file Presentation_1.pdf]

Supplementary Material

Phylogeny trumps chemotaxonomy: a case study involving *Turicella otitidis*

Inwoo Baek, Mincheol Kim, Imchang Lee, Seong-In Na, Michael Goodfellow and Jongsik Chun*

*** Correspondence:** Professor Jongsik Chun: Email: jchun@snu.ac.kr

1 Supplementary Data

2 Supplementary Figures and Tables

2.1 Supplementary Figures

Supplementary Figure 1. Genome-based phylogenetic tree reconstructed by PhyloPhlAn software for *T. otitidis* and neighboring species. Bar in the below signifies substitution rate per site. Blocks in column plots on the right side indicate the existence of each gene (or gene sets). Numbers at the nodes indicate support values calculated by FastTree.

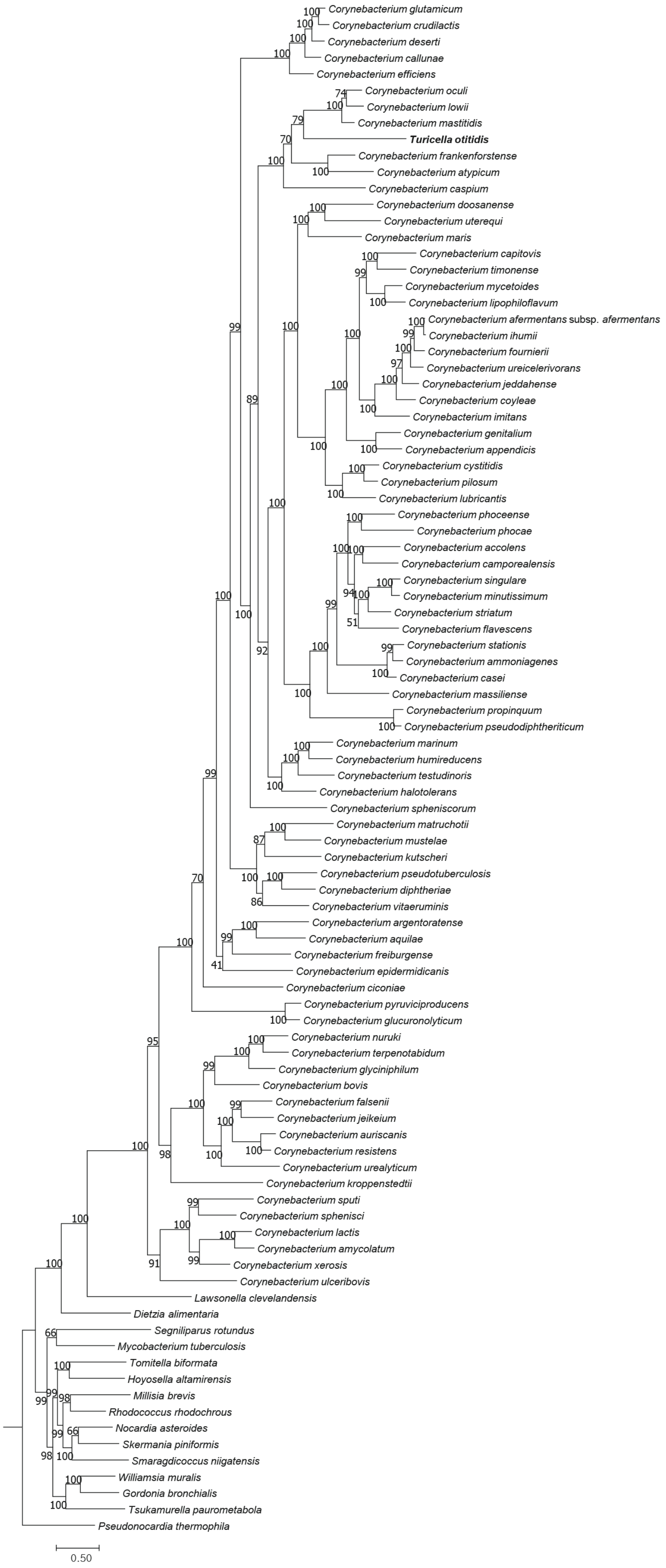
Corynebacterium glutamicum
Corynebacterium crudilactis
Corynebacterium deserti
Corynebacterium callunae
Corynebacterium efficiens
Corynebacterium oculi
Corynebacterium lowii
Corynebacterium mastitidis
Turicella otitidis
Corynebacterium frankenforstense
Corynebacterium atypicum
Corynebacterium caspium
Corynebacterium doosanense
Corynebacterium uterequi
Corynebacterium maris
Corynebacterium capitovis
Corynebacterium timonense
Corynebacterium mycetoides
Corynebacterium lipophiloflavum
Corynebacterium afermentans subsp. afermentans
Corynebacterium ihumii
Corynebacterium fournierii
Corynebacterium ureicelerivorans
Corynebacterium jeddahense
Corynebacterium coyleae
Corynebacterium imitans
Corynebacterium genitalium
Corynebacterium appendicis
Corynebacterium cystitidis
Corynebacterium pilosum
Corynebacterium lubricantis
Corynebacterium phoceense
Corynebacterium phocae
Corynebacterium accolens
Corynebacterium camporealensis
Corynebacterium singulare
Corynebacterium minutissimum
Corynebacterium striatum
Corynebacterium flavescens
Corynebacterium stationis
Corynebacterium ammoniagenes
Corynebacterium casei
Corynebacterium massiliense
Corynebacterium propinquum
Corynebacterium pseudodiphtheriticum
Corynebacterium marinum
Corynebacterium humireducens
Corynebacterium testudinoris
Corynebacterium halotolerans
Corynebacterium spheniscorum
Corynebacterium matruchotii
Corynebacterium mustelae
Corynebacterium kutscheri
Corynebacterium pseudotuberculosis
Corynebacterium diphtheriae
Corynebacterium vitaeruminis
Corynebacterium argentoratense
Corynebacterium aquilae
Corynebacterium freiburgense
Corynebacterium epidermidicanis
Corynebacterium ciconiae
Corynebacterium pyruviciproducens
Corynebacterium glucuronolyticum
Corynebacterium nuruki
Corynebacterium terpenotabidum
Corynebacterium glyciniphilum
Corynebacterium bovis
Corynebacterium falsenii
Corynebacterium jeikeium
Corynebacterium auriscanis
Corynebacterium resistens
Corynebacterium urealyticum
Corynebacterium kroppenstedtii
Corynebacterium sputi
Corynebacterium sphenisci
Corynebacterium lactis
Corynebacterium amycolatum
Corynebacterium xerosis
Corynebacterium ulceribovis
Lawsonella clevelandensis
Dietzia alimentaria
Segniliparus rotundus
Mycobacterium tuberculosis
Tomitella biformata
Hoyosella altamirensis
Millisia brevis
Rhodococcus rhodochrous
Nocardia asteroides
Skermania piniformis
Smaragdicoccus niigatensis
Williamsia muralis
Gordonia bronchialis
Tsukamurella paurometabola
Pseudonocardia thermophila
0.50

2.2 Supplementary Tables

Supplementary Table 1. UniProt database (http://www.uniprot.org/) accession numbers of genes used as the reference in the analysis. The abbreviation of the organism for each protein sequence is annotated in the parenthesis. Only protein sequences whose accession numbers in bold text are included in SwissProt. Organisms: ACE, *Acidothermus cellulolyticus* 11B; BH, *Bacillus halodurans* C-125; CG, *Corynebacterium glutamicum* ATCC 13032; CJ, *Campylobacter jejuni* subsp. *jejuni* serotype O:23/36; MT, *Mycobacterium tuberculosis* H37Rv; SCO, *Streptomyces coelicolor* A3(2); TT, *Thermus thermophilus* HB8.

Supplementary Table 2. Gene detected in *Corynebacterium* genomes associated with the biosynthesis of mycolic acids, and menaquinones. Numbers indicate sequence identity obtained by BLASTX alignment. Data were taken from the EzBioCloud database. Abbreviations of genes are listed in the below. ND, not detected.

Supplementary Table 3. Gene detected in *Corynebacterium* genomes associated with the biosynthesis of mycolic acids, and menaquinones. Numbers indicate bit score obtained by BLASTX alignment. Data were taken from the EzBioCloud database. Abbreviations of genes are listed in the below. ND, not detected.
